# Actin stabilizing compounds show specific biological effects due to their binding mode

**DOI:** 10.1038/s41598-019-46282-w

**Published:** 2019-07-05

**Authors:** Shuaijun Wang, Alvaro H. Crevenna, Ilke Ugur, Antoine Marion, Iris Antes, Uli Kazmaier, Maria Hoyer, Don C. Lamb, Florian Gegenfurtner, Zane Kliesmete, Christoph Ziegenhain, Wolfgang Enard, Angelika Vollmar, Stefan Zahler

**Affiliations:** 1 Department of Pharmacy, Ludwig-Maximilians-Universität, 81377 Munich, Germany; 2 Department of Chemistry, Center for Nanoscience (CeNS), Center for Integrated Protein Science Munich, (CIPSM) and Nanosystems Initiative Munich (NIM), Ludwig-Maximilians-Universität, 81377 Munich, Germany; 3 Center for Integrated Protein Science at the Department for Biosciences, Technical University Munich, 85354 Freising, Germany; 4 Institute for Organic Chemistry, Saarland University, 66123 Saarbrücken, Germany; 5 Department Biology II, Anthropology and Human Genomics, Ludwig-Maximilians-University, 82152 Martinsried, Germany; 6 Present Address: Instituto de Tecnologia Química e Biológica António Xavier, Universidade Nova de Lisboa, Nova de Lisboa, Portugal

**Keywords:** Computational chemistry, Cytoskeleton

## Abstract

Actin binding compounds are widely used tools in cell biology. We compare the biological and biochemical effects of miuraenamide A and jasplakinolide, a structurally related prototypic actin stabilizer. Though both compounds have similar effects on cytoskeletal morphology and proliferation, they affect migration and transcription in a distinctive manner, as shown by a transcriptome approach in endothelial cells. *In vitro*, miuraenamide A acts as an actin nucleating, F-actin polymerizing and stabilizing compound, just like described for jasplakinolide. However, in contrast to jasplakinolide, miuraenamide A competes with cofilin, but not gelsolin or Arp2/3 for binding to F-actin. We propose a binding mode of miuraenamide A, explaining both its similarities and its differences to jasplakinolide. Molecular dynamics simulations suggest that the bromophenol group of miurenamide A interacts with residues Tyr133, Tyr143, and Phe352 of actin. This shifts the D-loop of the neighboring actin, creating tighter packing of the monomers, and occluding the binding site of cofilin. Since relatively small changes in the molecular structure give rise to this selectivity, actin binding compounds surprisingly are promising scaffolds for creating actin binders with specific functionality instead of just “stabilizers”.

## Introduction

Actin is the most abundant protein in eukaryotic cells and plays a central role in many processes such as cell migration, cell division and intracellular transport. The discovery of actin binding compounds (cytochalasin D 1971^1^, phalloidin 1975^2^, latrunculin 1983^3^, jasplakinolide 1994^4^) has immensely fueled our knowledge about the biology of actin, and the natural compounds mentioned above have become standard tools in cell biology. To date, small actin-binding molecules are roughly divided into two groups: destabilizers (like e.g. latrunculin or cytochalasin D), and stabilizers (like e.g. phalloidin or jasplakinolide). However, during the past years, we have learned that the complexity of actin biology goes far beyond the regulation of overall polymerization and depolymerization^5^: actin does not merely form polymers with other actin molecules and subsequently depolymerize again. Rather, actin binding proteins continuously compete with each other for binding sites on actin (e.g. thymosin beta4 with profilin^6^, or MRTF with G-actin^7^) and with actin itself ^5^. This complex network allows for subtle control of the “actin-interactome” and related cell functions. Small actin-binding molecular compounds have been found to, in turn, compete with specific actin-binding proteins. For example, kabiramide C has been shown to compete with actin capping proteins like gelsolin in a kind of “molecular mimicry”^8^. This aspect of the interaction between small molecular inhibitors and actin has been largely neglected during their discovery and characterization. Therefore, the effects of actin-binding compounds on the cellular level (where the complex nature of the cytoplasm has to be considered) might, at least in part, be caused by mechanisms that have not yet been thoroughly considered.

The myxobacterial compound miuraenamide A (MiuA) is structurally related to the classical actin stabilizer jasplakinolide. Accordingly, it is has been presumed to also be an actin filament stabilizing agent^9,10^. Our in depth characterization of MiuA shows many similarities to jasplakinolide on a cellular and *in vitro* level. However, in the context of protein-protein interactions and transcriptional regulation MiuA shows striking functional differences to jasplakinolide, which can be in part explained by the unique binding mode we suggest.

## Results

### MiuA inhibits proliferation of endothelial cells at nanomolar concentrations and leads to actin aggregation

**Figure 1:**
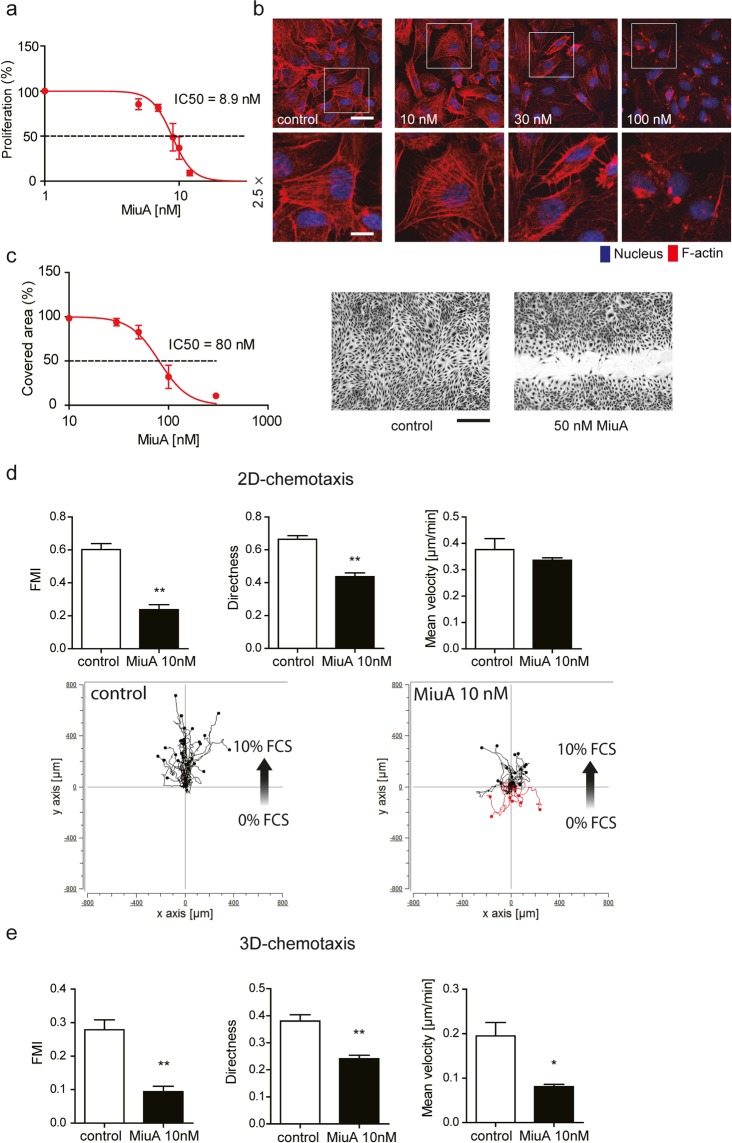
MiuA inhibits proliferation of HUVEC cells, induces actin aggregation and inhibits migration. (**a**) Half inhibitory concentration (IC_50_) of MiuA on proliferation (n = 3). (**b**) Actin morphology of HUVEC cells after treatment with the indicated concentrations of MiuA for 1 h: F-actin (phodamine phalloidin, red) and nuclei (Hoechst, blue). Representative images of 3 independent experiments performed in triplicates are shown. Upper panel: scale bar represents 75 µm, lower panel: zoomed sections from images above, scale bar represents 30 µm. The white frame indicates the zoomed in area. (**c**) Confluent HUVECs were scratched and treated with MiuA (indicated concentrations). After 16 h, images were collected and the cell covered area was analyzed. Left panel: the measured IC_50_ of MiuA from a scratch assay is shown (n = 3). Right panel: Representative images of the scratch assay. The scale bar represents 200 µm. MiuA inhibits endothelial cells migration (**d**) in 2D- and (**e**) in 3D-chemotaxis assays. Quantitative evaluation of the parameters X-Forward migration index, directness and mean velocity are shown (n = 3). Analysis of one representative experiment (out of triplicates in three independent experiments) is shown. (Kruskal– Wallis test with Dunns test as post hoc, *P < 0.05, **P < 0.01 vs. control, n = 3).

To assess the effect of MiuA on cells, we first measured proliferation. At nanomolar concentrations, MiuA inhibited the proliferation of HUVEC (human umbilical vein endothelial cells) with an IC_50_ value of around 9 nM (Fig. 1a), which is comparable to that in tumor cell lines (HCT116, HepG2, HL-60, U-2OS)^9^. Phalloidin staining of F-actin revealed that one hour of incubation with 30 nM of MiuA reorganizes the actin cytoskeleton into clusters or aggregates (Fig. 1b). At 100 nM MiuA, the actin cytoskeleton was completely destroyed and perinuclear actin aggregates were observed (Fig. 1b).

### MiuA inhibits HUVEC cell migration

To examine the effect of MiuA on the ability of cells to migrate, we performed a scratch assay. The migration of HUVEC cells was inhibited by MiuA in a concentration-dependent manner with an IC_50_ of approximately 80 nM (Fig. 1c). Since migration normally occurs in a gradient of growth factors, we studied the effect of MiuA on the chemotactic properties of HUVECs. In a 2D-chemotaxis assay, the directed migration of HUVECs was inhibited by 10 nM MiuA, as seen by the reduction of the forward migration index (FMI) and directness, while the mean velocity of movement was not affected (Fig. 1d). In 3D-chemotaxis, at 10 nM MiuA, cell migration directness, mean velocity and FMI were all significant reduced (Fig. 1e). These results indicate that MiuA has an overall effect on both cell motility and directionality of the movement, both in a simple 2D and in more physiological 3D environments. The reduced velocity in the 3D system could be explained by the cells having a reduced ability to squeeze through a small meshwork, as we have previously described^11^.

Overall, the cellular response to MiuA was typical for actin nucleating compounds^12^.

### MiuA and jasplakinolide differentially regulate gene expression

Treatment of HUVECs with a well tolerated concentration of MiuA (60 nM) or and equipotent concentration of jasplakinolide (120 nM) for 4 h caused dramatic effects on transcriptional regulation. With MiuA 779 genes were significantly regulated, with jasplakinolide 224 genes compared to untreated control (FDR < 0.1) (Table 1). The direct comparison between the two treatments revealed 101 significantly differentially expressed genes (Supplementary Table 1). When the regulated genes are classified into functionally relevant gene ontologies (Supplementary Table 2 for MiuA and Supplementary Table 3 for jasplakinolide), it becomes clear that most of the differences between miuA treatment and jasplakinolide do not result from different extents of regulation of the same genes, but from regulation of different sets of genes. Differentially expressed genes between the two conditions were used for gene set enrichment analysis. The 20 most significantly enriched biological processes can be seen in Table 2. 12 of these are unique for this comparison (highlighted in grey) and are not affected by either treatment alone vs. control.

**Table 1:** Gene regulation by MiuA (60 nM) and jasplakinolide (120 nM) after 4 h.

Treatment	Total number of differentially regulated genes	Number of upregulated genes	Number of downregulated genes
MiuA vs. control	779	384	395
Jasplakinolide vs. control	224	132	92
MiuA vs. Jasplakinolide	101	55	46

**Table 2:** Significant gene set enrichment (category “biological process”, Fisher’s exact test: < 0.01) based on differentially regulated genes by MiuA compared to jasplakinolide.

GO, ID	Term	Annotated	Significant	Expected	Fisher
GO:0071456	**cellular response to hypoxia**	146	7	1,09	0,00011
GO:0030509	BMP signaling pathway	90	7	0,67	0,00015
GO:0030900	**forebrain development**	213	8	1,6	0,00018
GO:0045446	endothelial cell differentiation	74	5	0,55	0,00022
GO:0007568	**aging**	194	7	1,45	0,0006
GO:0043280	**positive regulation of cysteine-type endopeptidase activity involved in apoptotic process**	92	5	0,69	0,00061
GO:0045666	positive regulation of neuron differentiation	202	7	1,51	0,00077
GO:0043410	**positive regulation of MAPK cascade**	272	8	2,04	0,00094
GO:0006935	**chemotaxis**	295	8	2,21	0,00158
GO:0032496	**response to lipopolysaccharide**	177	6	1,33	0,00207
GO:0002064	epithelial cell development	124	5	0,93	0,00233
GO:0001558	regulation of cell growth	260	7	1,95	0,00325
GO:0071902	**positive regulation of protein serine/threonine kinase activity**	198	6	1,48	0,00361
GO:0051051	**negative regulation of transport**	265	7	1,99	0,00361
GO:0072593	reactive oxygen species metabolic process	149	7	1,12	0,00471
GO:0051216	cartilage development	110	6	0,82	0,00545
GO:0043405	**regulation of MAP kinase activity**	218	6	1,63	0,00576
GO:0071560	cellular response to transforming growth factor beta stimulus	155	5	1,16	0,00604
GO:0006979	**response to oxidative stress**	300	7	2,25	0,00706
GO:0051155	**positive regulation of striated muscle cell differentiation**	31	5	0,23	0,00727

The GOs marked in bold are unique for the comparison MiuA vs. jasplakinolide and do not show up in MiuA or jasplakinolide vs. control.

### Effects of MiuA on the binding of proteins to G-actin

**Figure 2:**
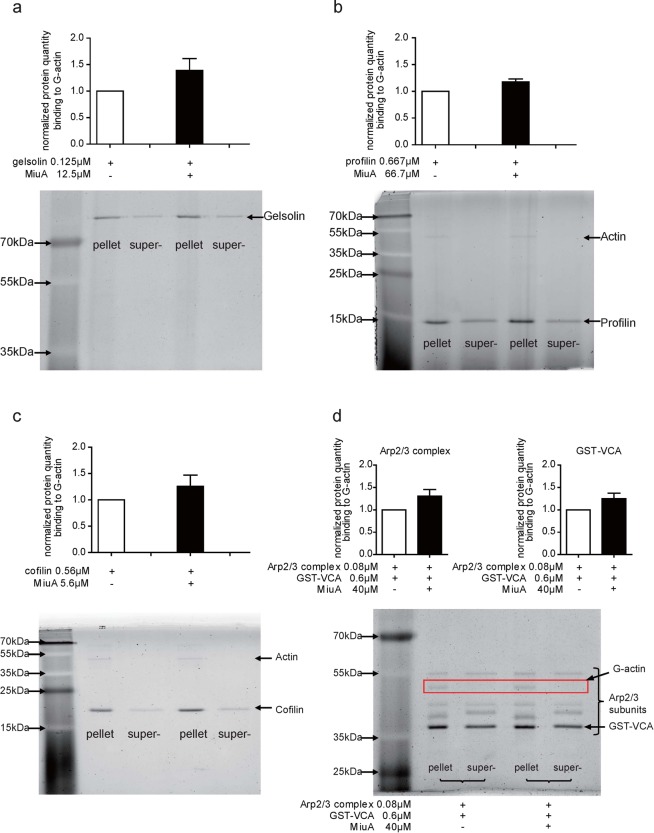
MiuA does not interfere with the binding of actin binding proteins to G-actin. G-actin beads were incubated with 0.01 mg/mL of the G-actin binding proteins (**a**) gelsolin, (**b**) profilin, (**c**) cofilin and (**d**) Arp2/3 complex & GST-VCA respectively, together with indicated concentrations of MiuA (molar ratio 1:10). The amount of G-actin binding protein in the pellets and supernatant was quantified. Representative images of protein bands and quantifications are shown. (Kruskal–Wallis test with Dunns test as post hoc, no significant differences vs. control, n = 3).

We next investigated whether of MiuA affects the binding of actin binding proteins (ABPs) to G-actin. To this end, we performed pulldown experiments with G-actin immobilized on beads and added single ABPs (gelsolin, profilin, cofilin and Arp2/3 complex with GST-VCA). As depicted in Fig. 2a–d, the total amount of the tested ABPs in G-actin pellets did not increase or decrease even if treated with a high amount of MiuA (50 or 100 fold excess), indicating that MiuA does not compete with gelsolin, profilin, cofilin or Arp2/3 complex for binding to G-actin. Detection of competitive effects in this assay was proven to be possible by showing the competition between cofilin and gelsolin (Supplementary Fig. 1c).

### In contrast to jasplakinolide, MiuA influences cofilin binding to F-actin

**Figure 3:**
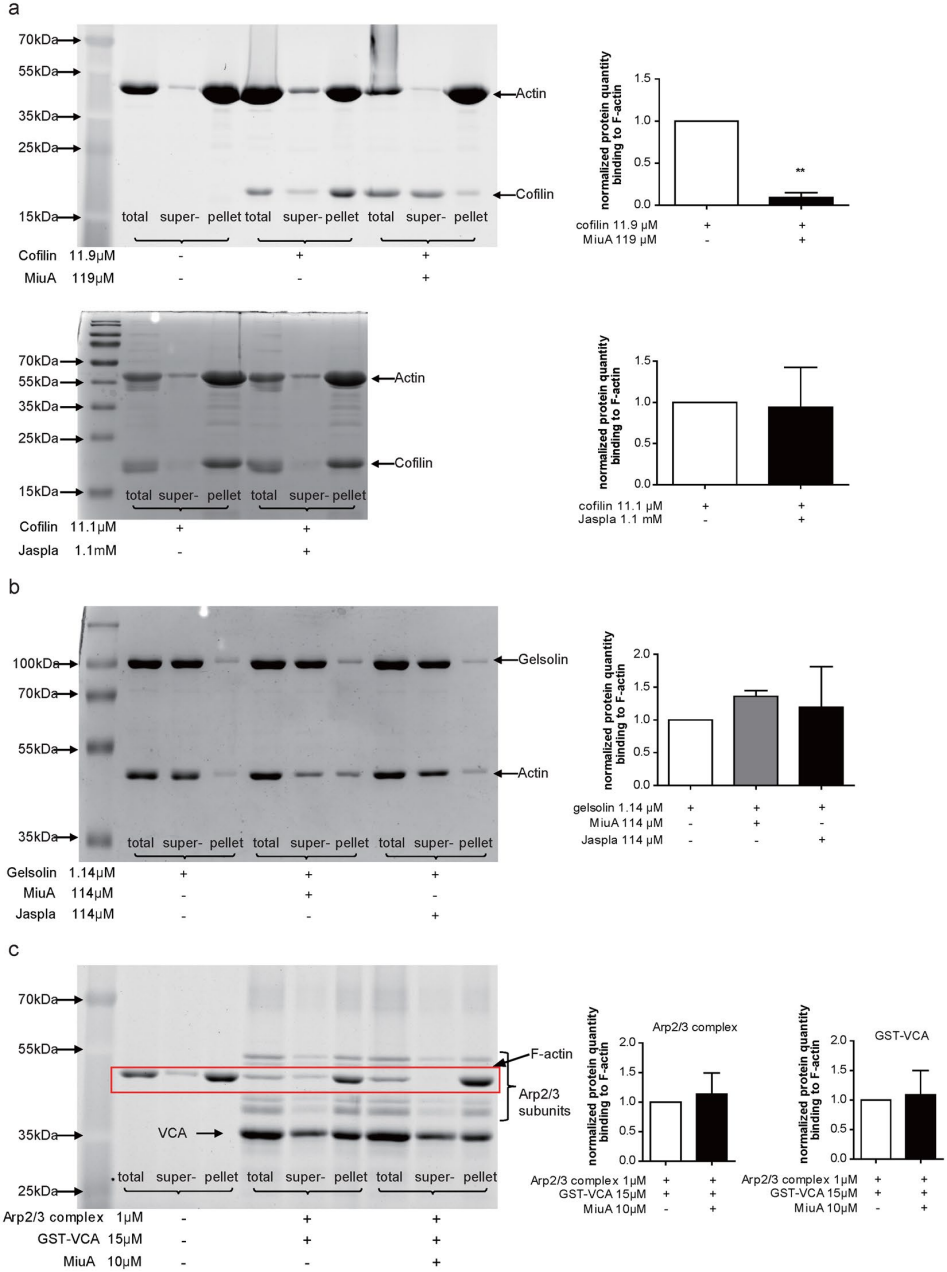
MiuA selectively inhibits binding of cofilin to F -actin. (**a**) MiuA inhibits binding of cofilin to F-actin (upper panel), while jasplakinolide does not (lower panel). F-actin was incubated with (**a**) cofilin, (**b**) gelsolin or (**c**) Arp2/3 complex & GST-VCA, together with MiuA or jasplakinolide (molar ratio 1:10). Binding of gelsolin (**b**) or Arp2/3 (**c**) to F-actin were not influenced by MiuA or jasplakinolide. The amount of F-actin binding protein in the total sample, supernatant and pellets was quantified. Representative images of protein bands and quantifications are shown. (Kruskal–Wallis test with Dunns test as post hoc, **P < 0.01 vs. control, n = 3).

To investigate potential competition of MiuA and ABPs for binding to F-actin, we also performed a binding assay with cofilin, gelsolin and Arp2/3 complex (together with GST-VCA) to F-actin. In the absence of MiuA, cofilin was largely found in the F-actin pellet (Fig. 3a), as expected from the known interaction between cofilin and F-actin. In the presence of MiuA, a significant reduction of cofilin was observed in the pellet (Fig. 3a, upper panel). Jasplakinolide did not change binding of cofilin to F-actin, even when added at a ten-fold concentration in comparison to MiuA. (Fig. 3a, lower panel). Neither MiuA, nor jasplakinolide seem to influence binding of gelsolin (Fig. 3b) to F-actin. However, it has to be taken into account that gelsolin has an F-actin severing activity, which shows by a shift of actin into the supernatant fraction (Fig. 3b). Thus, the experimental situation is quite complex and we can only comment on the binding of gelsolin to the F-actin remaining in the pellet fraction. MiuA did not affect Arp2/3 complex binding to F-actin, since the amounts of Arp2/3 complex in F-actin pellet were similar in the presence or absence of MiuA (Fig. 3c). These results suggest that MiuA selectively interferes with the binding of cofilin to F-actin, and that this action is specific for MiuA, since it was not mimicked by jasplakinolide. The inhibition of cofilin binding to F-actin by MiuA also seems to work in the cellular context, since treatment of HUVECs with 50 nM MiuA for 30 min reduced co-localization of cofilin with F-actin, as shown by confocal microscopy (Supplementary Fig. 2).

### Miuraenamide A induces actin nucleation and polymerization, as well as stabilization of filaments

**Figure 4:**
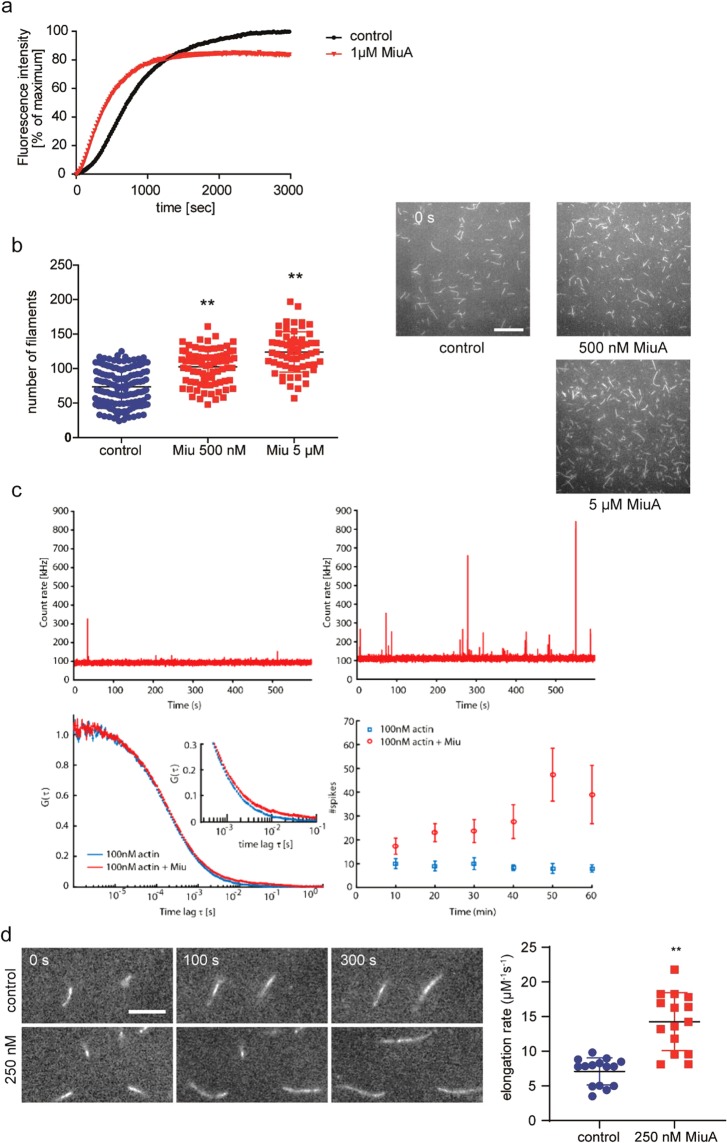
MiuA enhances actin polymerization and nucleation. (**a**) The pyrene assay shows an accelerated polymerization of actin after treatment with MiuA. (**b**) The TIRF assay shows increased number of filaments indicating more nucleation of actin upon addition of MiuA. 138 frames (control), 81 frames (MiuA 500 nM), and 60 frames (MiuA 5 µM) respectively from 3 independent experiments were evaluated. In (**a**) and (**b**) the final concentration of G-actin was 1 µM. (**c**) Measurement of actin nucleation by FCS: The fluorescence signal of 100 nM actin-atto488 as a function of time after 1 h incubation, without (upper left panel) and with (upper right panel) 10x molar excess of MiuA. Lower left: FCS curves of 100 nM actin-atto488 with and without MiuA. Inset: detail of the autocorrelation function at longer timescales showing the formation of larger oligomers with MiuA. Lower right: Number of spikes due to filaments diffusing through the observation volume in 10 min time intervals. The data show that up to 6 times more spikes are observed during the course of one hour in the presence of MiuA. Error bars represent the standard error of the mean of three independent measurements. (**d**) Actin elongation measured using TIRF microscopy. Left panel: Representative time series of fluorescence images show the elongation of actin filaments. Right panel: The calculated actin elongation rates from 15 control and 15 MiuA treated filaments (n = 3 independent experiments). Scale bar in (**b**) represents 15 µm. Scale bar in (**d**) represents 5 µm. (Kruskal–Wallis test with Dunns test as post hoc, **P < 0.01 vs. control).

Based on previous findings^10^, we investigated the molecular mechanism of the interaction of miuraenamide A (MiuA) with actin alone. First, we examined the effect of MiuA on the actin polymerization process in a pyrene assay. With 1 μΜ MiuA, actin nucleation and polymerization was faster (Fig. 4a) but, interestingly, peak fluorescence was lower. This might result from substrate consumption by the rapid formation of small actin aggregates. Monitoring actin filament assembly by TIRF (total internal reflection fluorescence) microscopy showed that MiuA increased the overall rate of formation of actin filaments (Fig. 4b). This increase in the number of filaments formed over time suggested a stabilizing effect during nucleation. We also monitored the formation of oligomers by Fluorescence Fluctuation Spectroscopy^13^. The appearance of spikes in the data represent stabilized oligomers^13^. Addition of MiuA to actin below the critical concentration for filament assembly (c_c_ = 200 nM) showed an increase in the appearance and size of large oligomers over 1 h (Fig. 4c). To investigate the formation of small oligomers, we calculated the autocorrelation function in regions where no spikes were observable. The appearance of a second diffusing species with a significantly longer diffusion time indicates formation of small nuclei with MiuA (Fig. 4c). These results suggest that MiuA favors polymerization by stabilizing the early stage oligomers formed during nucleation.

**Figure 5:**
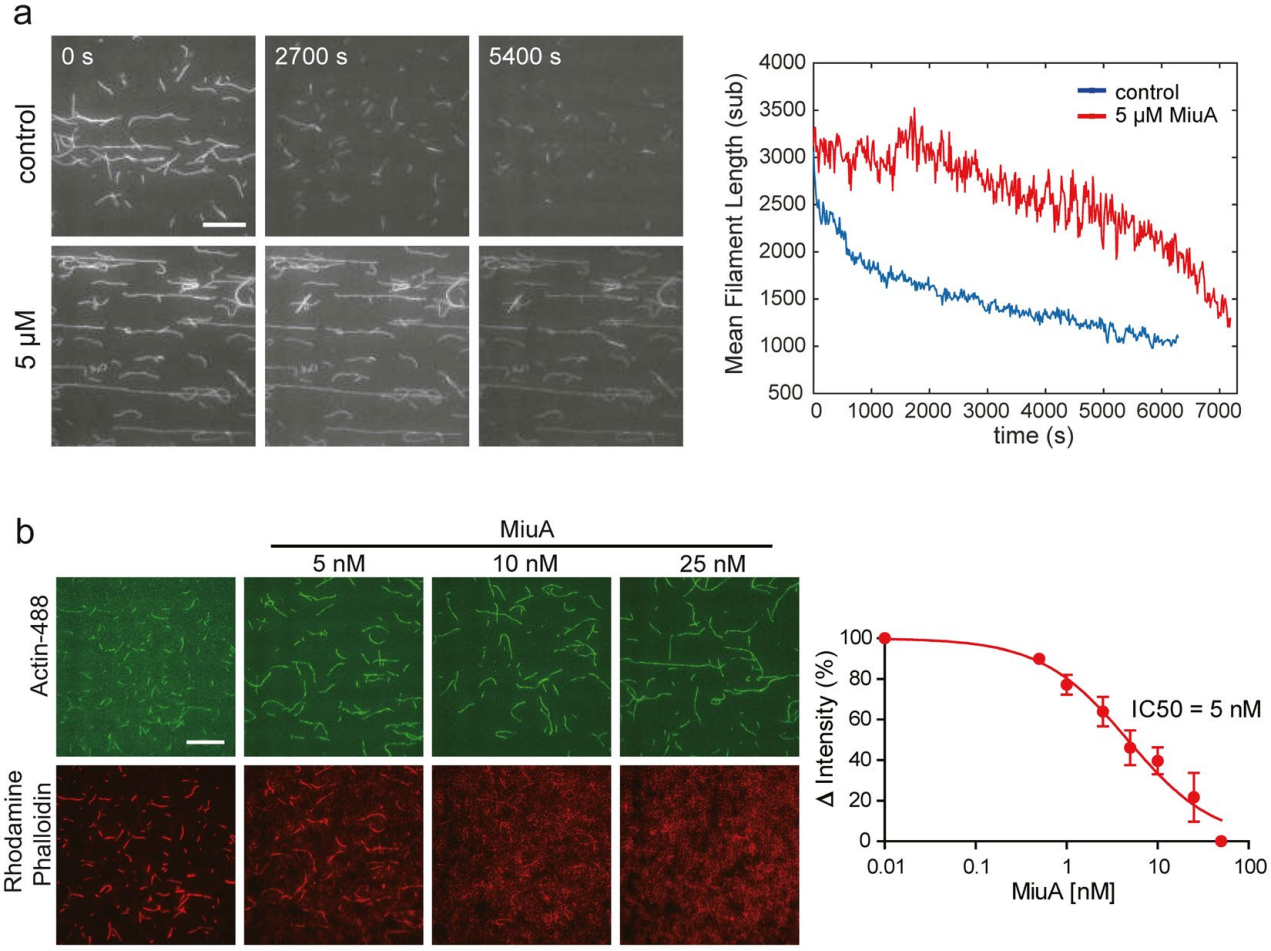
MiuA stabilizes F-actin and competes with phalloidin for binding to F-actin. (**a**) MiuA decelerates depolymerization of F-actin as shown by TIRF microscopy. Left panel: Representative fluorescence images at different time points during F-actin depolymerization. Right panel: The average length of actin filaments was quantified as depolymerization parameters. Scale bar: 15 µm. (**b**) MiuA competes with phalloidin for binding to actin filaments. Atto488 labeled F-actin was incubated with 16.5 nM rhodamine phalloidin and increasing concentrations of MiuA. Images of actin filaments were taken by TIRF microscopy. Left panel: TIRF images showing the Atto488-labeled actin filaments (upper panels) and rhodamine-labeled phalloidin (lower panels). At increasing concentrations of MiuA, the binding of phalloidin to actin filaments decreased. Right panel: The red fluorescence of actin filaments was quantified and an IC_50_ of MiuA was calculated (n = 3). Scale bar: 15 µm.

Next, we investigated the influence of MiuA on the rate of actin filament elongation using TIRF microscopy. In the absence and presence of 250 nM of MiuA, we monitored the length of individual filaments as a function of time and found that the elongation rate doubled compared to control samples (Fig. 4d). To determine whether MiuA plays any role in stabilizing actin filaments, we monitored the disassembly of labeled actin filaments in the presence and absence of 5 μM MiuA by TIRF microscopy. As expected, the length of actin filaments decreased in a time-dependent manner in the absence of MiuA. In the presence of MiuA, disassembly of filaments was retarded (Fig. 5a). These results indicate that the binding of MiuA promotes both, the assembly and stabilization of actin filaments.

### MiuA competes with phalloidin for binding to F-actin

Phalloidin is a classical F-actin stabilizing compound with a well characterized binding site^14^. To investigate whether MiuA binding to actin competes with phalloidin binding, we performed a competition assay with rhodamine labeled phalloidin. Rhodamine-labeled phalloidin could be displaced from actin filaments by MiuA in a concentration dependent manner (Fig. 5b) with an IC_50_ of about 5 nM (Fig. 5b, right panel). This indicates that MiuA may share a proximal binding site with phalloidin on F-actin, or that MiuA allosterically influences binding of phalloidin.

### MiuA tightens actin monomer assembly and hinders binding of cofilin by shifting the D-loop

**Figure 6:**
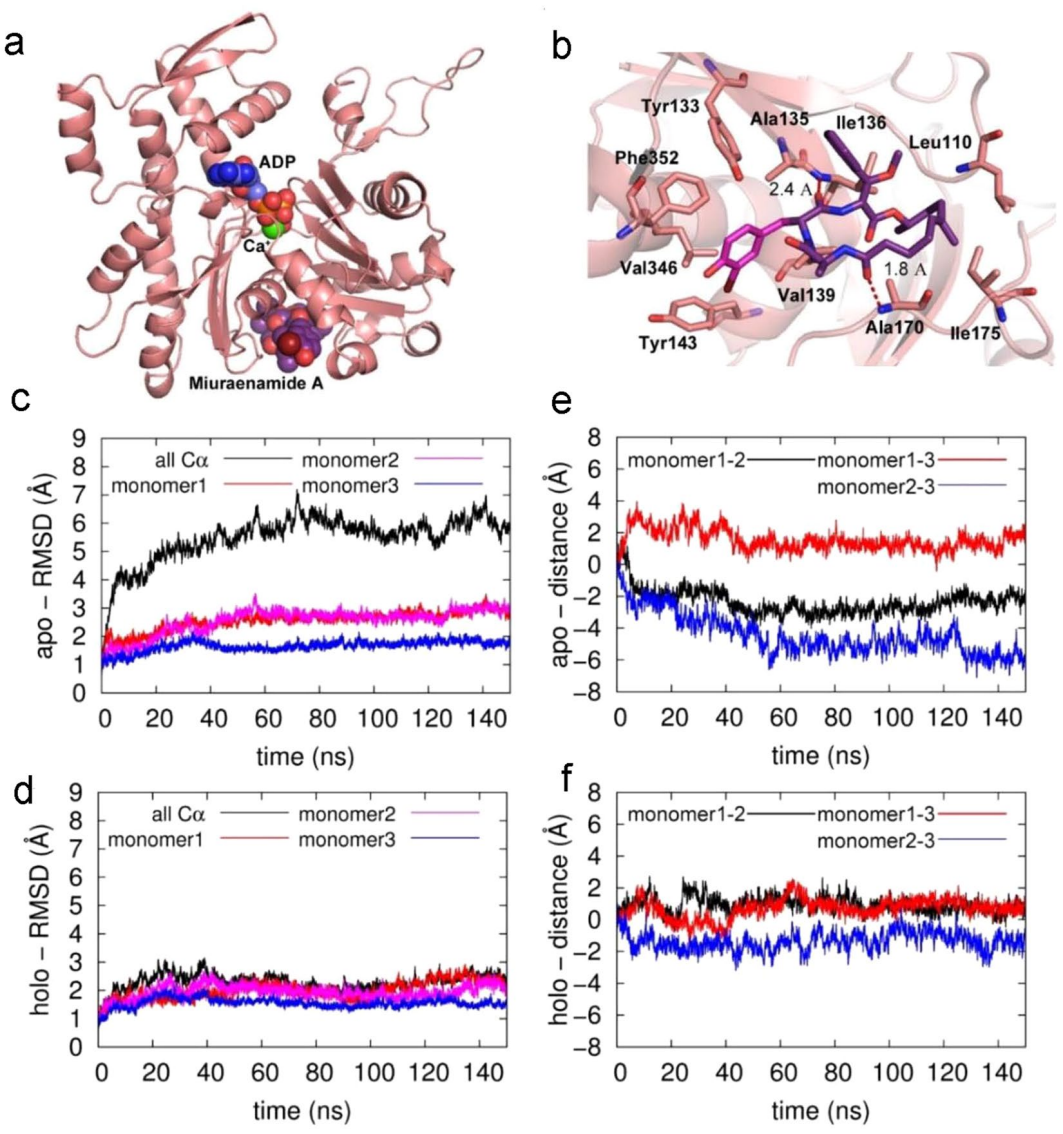
Structures of the docked Miuraenamide A and after 25 ns of MD equilibration. A global view of the system is presented in (**a**,**b**) gives a detailed view of the binding site. Actin is shown in cartoon representation, ADP (blue), Ca+ (green) and Miuraenamide A (purple) are shown as van der Waals spheres (**a**) and as sticks (**b**). Residues interacting with the ligand are shown with sticks. (C + D) RMSD time-series (Å) of the apo- (**c**) and holo- (miuraenamide A bound) (**d**) simulations. The black, red, magenta, and blue colors represent the deviations of α carbon atoms of the trimer, monomer1, monomer 2 and monomer 3 from the initial structures, respectively. (e + f) The deviation of the distances of pairs of monomers (based on the center of mass of the stable β-sheet regions of each monomer) from the initial structure as a function of time for the apo- (**e**) and holo- (**f**) systems.

**Figure 7:**
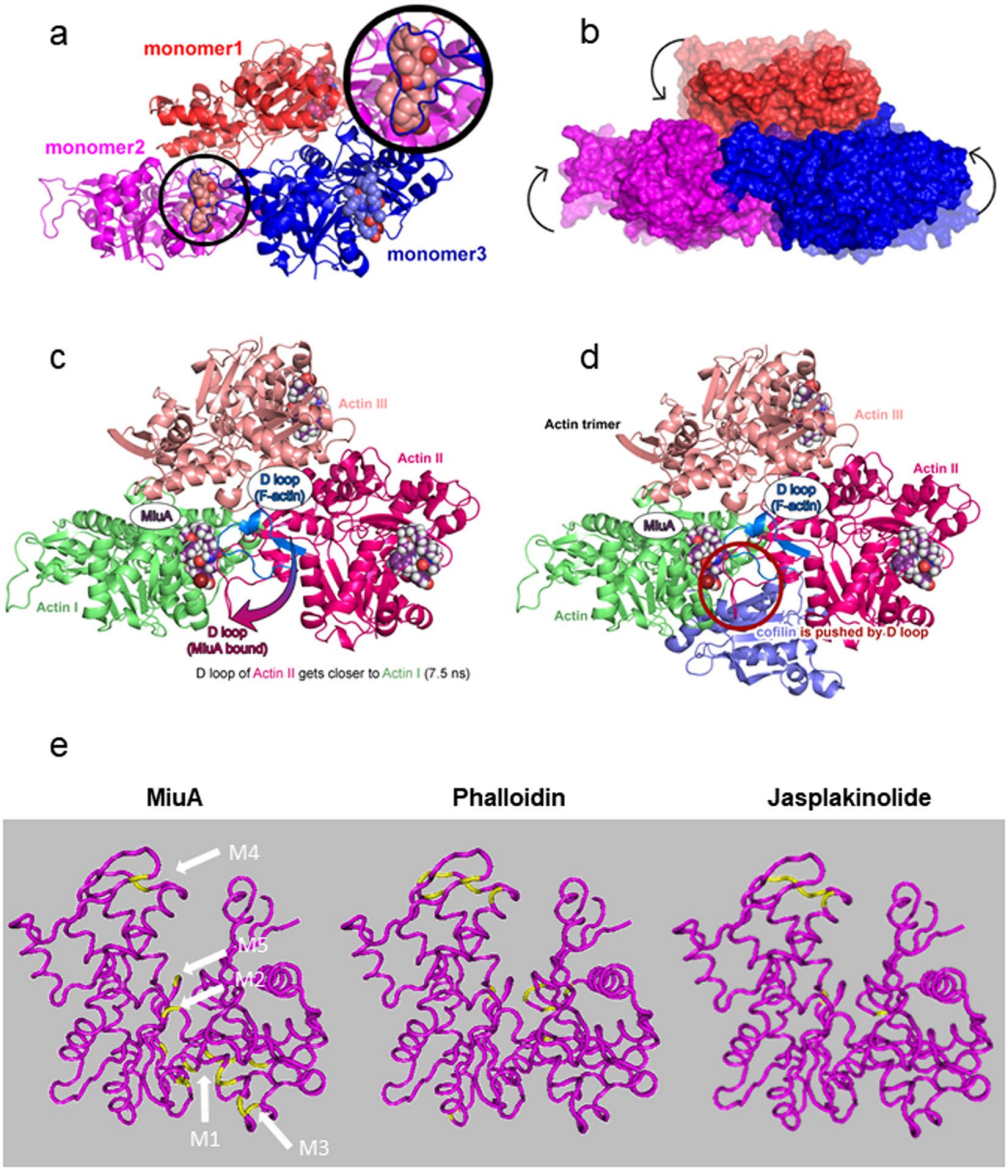
Model for the compaction of the actin trimer and for the replacement of cofilin by the movement of the D-loop. (**a**) The orientation of MiuA within the trimer is depicted with an additional focus on the interaction between the D-loop of monomer 3 and the ligand of monomer 2 in the inset. (**b**) The difference in packing between the apo- (transparent coloring) and holo- (solid coloring) trimer systems is illustrated for a representative structures of the two complexes. (**c**) Once MiuA binds to F-actin, the D-loop of monomer 2 moves closer to monomer 1 during the molecular dynamics simulation. (**d**) MiuA and cofilin bound to the F-actin (the position of cofilin is shown as bound in the apo-structure with F-actin). The new conformation of the D-loop overlaps with the bound conformation of cofilin. (**e**) Actin binding sites for miuA, phalloidin and jasplakinolide. Five potential binding sites for MiuA (M1 to M5) obtained by the computer-based docking simulations are projected into the G-actin monomer (PDB: 3HBT). G-actin: magenta, respective binding sites of the other compounds according to literature: yellow.

Since the binding mode of MiuA is still unknown, we investigated the atomic details of possible docking sites. Five possible binding sites for MiuA were suggested from the docking computations (Fig. 6a) performed for the actin monomer. The binding free energies of MiuA within each binding site (M1 to M5) were found as: −39.4 kcal/mol, −35.3 kcal/mol, −19.1 kcal/mol, −21.9 kcal/mol and −29.3 kcal/mol, respectively. M1 lies in the hydrophobic cleft of the actin monomer (hydrophobic interactions with Leu110, Ile136, Ile175, Val139, and Ala170 of actin, as well as Tyr133, Phe352, Tyr143). M2 is at Lys336, M3 at Leu349 and Ile345, and M5 at Asp157. Interestingly, M4 is at Phe200 and Leu242, two binding sites for phalloidin^14^ and jasplakinolide^15^, respectively. For further computational studies we focused on M1, the energetically most favorable site (Fig. 6b,c). The amide oxygen atoms of the ligand form hydrogen bonds with the backbone nitrogen atoms of Ala135 and Ala170. These hydrogen bonds resemble those generally observed in secondary structure interactions of proteins, highlighting the peptidomimetic character of MiuA. There is a unique interaction between the macrocycle and the protein, in which three aromatic residues from actin (Tyr133, Phe352, Tyr143) and bromophenol of the MiuA form an aromatic cyclic tetramer. To assess the influence of the docked MiuA on the stability and structure of F-actin, the equilibrated (25 ns) monomer structure was placed into an F-actin trimer scaffold structure for molecular dynamics simulations. Figure 6d,f show that for the MiuA-bound holo-complex a stable complex structure is obtained after a short equilibration time of about 20 ns, which is structurally very similar to the original build model with RMSD (root-mean-square-deviation) values oscillating around 2 Å. For the apo-complex a longer equilibration period of about 60 ns is needed until a stable equilibrated structure is obtained (i.e. the RMSD values remain stable). This can be attributed to the fact that the final apo-structure shows larger structural deviations from the original model (overall Cα-RMSD around 6 Å, Fig. 6c black line). A more detailed analysis shows that the structural changes within the individual monomers remain very small (Cα-RMSD around 2 Å, Fig. 6c blue/red/magenta lines), but that there is an overall shift in the relative position of the monomers with respect to each other within the first 60 ns of the simulation (Fig. 6e). The latter can be directly observed by monitoring the distances between the center-of-masses of the individual monomer pairs (Fig. 6e). The largest movement occurs between monomers 2 and 3, whereas the distances between monomers 1–3 and 1–2 remain relatively stable. After 60 ns all distances and thus monomer positions remain stable, indicating that a stable, equilibrated apo-complex structure is reached. In the holo-structure the D loop of monomer 3 moves towards MiuA (Fig. 7a) during equilibration, forming strong interactions with the ligand. This leads to stabilization and even tightening of the monomer 2–3 interface region and thus overall stabilization of the trimeric complex (Fig. 7b). In the apo-structure the corresponding loop movement does not occur, as no ligand is present, leading to the observed shift of the monomers with respect to each other. However, analysis of overall magnitude of these changes in the apo-complex (Fig. 7b, shaded structure) and the comparison of the final structure to the original scaffold structure shows that the structural changes still remain within the experimental boundaries of the scaffold, indicating that a stable, realistic minimum was found for the apo-structure.

### Binding of cofilin to F-actin is inhibited by D-loop rearrangement

The rearrangement of the D-loop from monomer 3 explains the tighter binding between the actin monomers 2 and 3 as MiuA stabilizes these inter-strand contacts. To investigate whether this binding might impact the interaction with cofilin, we aligned our MiuA bound structure within the cofilin bound crystal structure (PDB:3J0S^16^, Fig. 7c,d). This structural alignment shows that the cofilin binding site is situated adjacent to the MiuA binding site. When both MiuA and cofilin attempt to bind to F-actin simultaneously, the changes in the conformation and location of the D-loop triggered by MiuA binding lead to a partial screening of the cofilin binding site by the D-loop. This might prohibit binding of the cofilin complex and explain why cofilin competes with MiuA for binding to actin.

## Discussion

Actin is the most abundant protein in eukaryotic cells and the protein with the greatest variety of binding partners^17–19^. Due to its ubiquitous expression and its many biological functions, actin has not been advanced as a clinically relevant drug target. The question how selective functional effects could be elicited by just increasing or decreasing overall actin polymerization by the use of small molecular binders in particular has precluded the use of actin targeting natural compounds in a therapeutic setting for many years in spite of their availability^1,3,4^. In recent years, however, it has become increasingly clear that actin-binding proteins (ABPs) are the key to localized and specific regulation of actin polymerization and depolymerization. For example, it has been shown that JMY or WHAMM are needed to localize actin nucleation specifically to the autophagosome^20,21^. Since the amount of G-actin in a cell is limited, a competition between actin monomers, F-actin, and ABPs for binding to the available G-actin pool takes place, and turns out to be relevant for regulation of actin function^5,18,22^. Exploiting this level of regulation by using the concept of “biomolecular mimicry”^8,23^ would open a new field of biological tools or even therapeutic options by developing novel and much more specific actin targeting compounds.

To date, actin binding compounds are largely classified as depolymerizers (destabilizers) or polymerizers (stabilizers). This second group consists of classical compounds like e.g. phalloidin, jasplakinolide and the chondramides, which have structural similarities and seem to share the same binding site on F-actin^24^. The myxobacterial compound miuraenamide A (MiuA)^25^ is also structurally related to these compounds, and has turned out to have a functional profile similar to phalloidin or jasplakinolide (enhanced polymerization and stabilization, cell morphology and cytotoxicity)^9,10^. We have previously used MiuA as a *bona fide* actin polymerizing compound in cellular studies, and have shown effects on cell migration and transcriptional activity that can be ascribed to its actin stabilizing nature^11,26^. In the present study we established dose response curves for MiuA on proliferation in primary endothelial cells (HUVECs), which are similar to those in tumor cell lines^9,11^, and lie in the same range as those for jasplakinolide^27^. Interestingly, MiuA inhibited migration of HUVECs in contrast to jasplakinolide, which has been previously reported to paradoxically increase migration^28,29^. When we compared the effects of MiuA on transcription with those of a cellularly equipotent concentration of jasplakinolide, we detected profound differences: 101 genes were regulated in a significantly different way. Among the most significantly influenced gene ontologies annotated to these genes were some, which might in part explain the differences in migration (e.g. chemotaxis, negative regulation of transport, regulation of MAP kinase activity). Actin is able to affect transcription in multiple ways^30^, most of which are based on interaction of actin binding proteins with actin. Indeed we found specific differences between MiuA and jasplakinolide concerning interaction of prototypic actin binding proteins with actin *in vitro*: while MiuA competed with cofilin for binding to F-actin (but not to G-actin), jasplakinolide did not. In contrast, gelsolin and Arp2/3 binding were influenced by neither compound, though they have binding sites similar to those of cofilin. Thus, there seems to be a considerable structural specificity of the inhibition.

When looking for a specific mode of action of MiuA on isolated actin in the TIRF assay and by FCS (fluorescence correlation spectroscopy), we found no surprises: nucleation and elongation were increased, F-actin was stabilized and MiuA competed with phalloidin for binding to F-actin, which indicates binding sites of both compounds in close proximity.

However, *in-silico* studies revealed that MiuA binds to motifs similar and close to, but still different to the binding site of phalloidin, chondramide, or jasplakinolide^24,31,32^. Interaction of a compound with F-actin is complex, since the compound may bind to three actin molecules at the same time at different sites. For the sake of simplicity, we collapsed all 5 binding sites of MiuA (M1 to M5), phalloidin and jasplakinolide on one monomer, respectively. This reveals that M4 is a binding site shared with phalloidin and jasplakinolide (which in part explains the competition with phalloidin), M2 and M5 also show similar binding sites as phalloidin and jasplakinolide, but M1 and M3 address sites in the actin molecule, which are not bound by the other F-actin stabilizing compounds. The mode of binding to this site is unique in that the bromophenol substitution of the macrocycle and the three aromatic residues from actin (Tyr133, Phe352, Tyr143) form an aromatic cyclic tetramer. Similar motives (involving more or less aromatic cycles) were shown to be frequently observed in proteins and contribute to the stabilization of their tertiary structures^33–35^. This relatively strong interaction (about 5 kcal/mol in vacuum^36,37^) reveals the importance of the bromophenol ring as a crucial moiety of the macrocyclic MiuA. The second phenyl ring bound to the macrocycle, on the other hand, is not engaged in any crucial interactions.

Analysis of the influence of MiuA binding on the F-actin structure by simulation of actin-trimer complexes with and without bound MiuA showed that the binding of MiuA influences the overall structures of the actin timer, as for the apo-actin-trimer a different relative arrangement of the individual actin monomers was observed compared to the holo-trimer complex. A more detailed analysis of the structures revealed that the MiuA ligand bound to the actin DNAse I binding site triggers the migration of the D-loop in monomer 3 towards actin. This phenomenon increases the contact area between the monomers. Presumably MiuA further acts as a buffer between these two monomers and stabilizes the compact trimer form. Comparisons between holo- and apo-structures show that the interaction between monomers 2 and 3 is affected most by the binding of MiuA, although all monomers contain a ligand in the holo-system. This is consistent with the fact that only the ligand of monomer 2 is located at an inter-monomeric interface in the trimer simulation. This observation, together with the RMSD analysis, further highlights that the binding of MiuA does not affect the individual integrity of the units but regulates the inter-monomeric interactions. Overall, MiuA binding ensures a tighter and stronger packing of the actin monomers compared to the apo F-actin by shifting the D-loop, which is indispensable for F-actin stabilization^31^.

Our molecular dynamics simulations also offer an explanation for the inhibition of cofilin binding by MiuA: the shift of the D-loop by MiuA leads to a clash between this loop and cofilin in its bound conformation.

The transcriptional differences between MiuA and jasplakinolide might in part be linked to the effects of MiuA on binding of cofilin to F-actin, since cofilin directly modulates nuclear architecture^38^, but also plays an important role in regulating nuclear actin levels^39^, which, in turn, have an influence on transcriptional regulation^40^. Our finding that the co-localization of cofilin and F-actin is decreased in cells treated with MiuA supports the hypothesis that this competition indeed occurs in the cellular context. However, due to the complexity of the role of actin and actin binding proteins in transcriptional regulation, we can not rule out other modes of action of MiuA treatment.

Though MiuA has been termed to be structurally similar to jasplakinolide, we identified a unique mode of action that selectively inhibits binding of cofilin to F-actin and causes transcriptional regulation different from that of jasplakinolide. Consequently, we reveal that small structural differences in actin binding compounds can cause functional selectivity, which was not presumed in this class of molecules. The much better synthetic accessibility of MiuA^9^, as compared to other actin stabilizing compounds, makes it an ideal candidate for studying structure-activity relationships. Ideally, it could be feasible to create derivatives that do not influence actin polymerization per se, but selectively modulate the binding of single actin-binding proteins, and circumvent the selectivity issues, which have hindered the development of actin-modulating drugs.

## Methods

### Compounds

Miuraenamide A (MiuA) was synthetized as described previously^9^. For the experiments MiuA was dissolved and stored in DMSO (dimethyl sulfoxide). Jasplakinolide was purchased from Santa Cruz (Heidelberg, Germany). For all experiments with compounds respective solvent controls with a matching concentration of DMSO were performed (termed “control”).

### Proliferation assay

HUVECs (human umbilical vein endothelial cells) were purchased from Promocell, Heidelberg, Germany, and cultured with endothelial growth medium (Promocell), supplemented with 10% FCS under constant humidity at 37 °C and with 5% CO_2_. Upon confluency, cells were split in a ratio 1:3 in T25 flasks. Cells were used for functional assays at passage 6. 1.5 × 10^3^ cells/well were seeded in 96-well plates. After 24 h, cells were treated with the indicated concentrations of MiuA and incubated for further 72 h. Subsequently, cells were stained with 100 μL/well Crystal Violet solution (0.5% Crystal Violet and 20% methanol in H_2_O) for 10 min, then washed with H_2_O and dried. Finally, 100 μL/well Na-citrate solution (50% ethanol, 50% 0.1 M Na-citrate in H_2_O) were added, incubated on a shaker for 5 min and measured at 550 nm using a microplate reader (Sunrise, Tecan, Maennedorf, Switzerland).

### Fluorescence imaging

HUVECs (1.5 × 10^3^ cells/well) were seeded in ibidi μ slides (ibidi GmbH, Munich, Germany), treated with MiuA as indicated for 1 h, and subsequently fixed with 4% (v/v) paraformaldehyde. F-actin was stained with rhodamine phalloidin (1:400, R 415, Molecular Probes, Life technologies) and nuclei were labeled with Hoechst stain (bisBenzimide H33342 trihydrochloride, B2261 Sigma-Aldrich). Images were taken using a Zeiss LSM 510 META confocal microscope (Zeiss, Jena, Germany). For the co-localization experiments cells were treated for 30 min with 50 nM MiuA, and subsequently stained as described above with the addition of an anti-cofilin antibody (D3F9, rabbit, Cell Signaling), and a subsequent step of incubation with an Alexa488 labelled secondary antibody (goat anti rabbit, A – 11008, Invitrogen). These images were obtained on an inverted SP8 confocal microscope (Leica, Germany).

### Scratch assay

Confluent HUVECs were scratched with a custom made tool and treated with the indicated concentrations of MiuA. Cells were allowed to migrate for 16–24 h, then washed with 100 μL/well PBS (including Mg^2+^, Ca^2+^), stained with 100 μL/well Crystal Violet solution for 10 min, washed with water, and dried. Images were collected using an inverted microscope (Zeiss Axiovert 100). Image analysis was performed with ImageJ. Migration was quantified as the percentage of cell-covered area compared with the total image area.

### 2D and 3D chemotaxis

For the 2D-chemotaxis assay, HUVECs (50 × 10^3^/well) were seeded in a μ-Slide Chemotaxis ibiTreat (ibidi GmbH, Munich, Germany), treated with the indicated concentrations of MiuA and incubated for 2 h. Then a gradient of FCS between 0 and 10% (v/v) was applied to the slide according to the manufacturer’s instructions. For the 3D-chemotaxis assay, HUVECs (50 × 10^3^/well) were seeded in matrigel (REF 356231, Corning, Matrigel® Basement Membrane Matrix Growth Factor Reduced, Phenol Red Free, *LDEV-Free) in a μ-Slide Chemotaxis3D ibiTreat, treated as indicated and incubated. After 0.5 h, a gradient FCS between 0 and 10% (v/v) was applied. Time lapse image sequences of cell migration were collected every 10 min for 20 h using an inverted Nikon microscope (Eclipse), equipped with a custom made incubation chamber. Images were analyzed using the Chemotaxis and Migration Tool (Version 2.0 with ImageJ Plugin). Cell mean velocity, directness and the X-forward migration index (FMI) were calculated.

### G-actin binding assay

All actin binding proteins were purchased from Hypermol, Bielefeld, Germany. They were either produced recombinantly (cofilin, profiling) or isolated from porcine tissue (gelsolin, Arp2/3). The G-actin binding assay was performed using the ‘Actin-Toolkit G-Actin Binding’ (Hypermol). In this kit, G-actin is coupled to SepharoseTM as G-actin beads. Binding of actin-binding proteins (ABPs) to G-actin is highly specific, and thus, the ABPs bound to G-actin are co-precipitated under low centrifugal forces. G-actin beads (1/4 volume of 1 tube) were pretreated with MiuA (20 μM) for 30 min at room temperature under agitation, then the respective ABPs were added, and incubated for 1 h at RT. After incubation, the sample was spun at 6,000 × g at 4 °C for 4 min and 40 μL supernatant were prepared for an SDS-gel. The G-actin beads were washed, boiled, and resuspended in 25 μL of 1 × SDS-sample buffer. Both, the supernatant and the G-actin beads were separately loaded onto a SDS-PAGE gel. After electrophoresis, the gel was imaged using a ChemiDoc Imaging System (Bio-Rad Laboratories, Munich, Germany). The amount of each protein was quantified using the Image Lab 6.0 Software. Since binding of gelsolin is Ca^2+^ dependent, the binding assay was carried out in the presence of 200 µM CaCl_2_ in this case. As control for unspecific binding of proteins, uncoupled beads were used (Supplementary Fig. 1a), gelsolin was used in the absence and presence of Ca^2+^ (Supplementary Figure 1a), and gelsolin and cofilin were used in a competition experiment (Supplementary Fig. 1b).

### F-actin binding assay

The F-actin binding assay was performed using the ‘Actin-Toolkit F-Actin Binding’ (Hypermol, Bielefeld, Germany). F-actin was prepared by polymerizing G-actin with polymerization buffer for 30 min at room temperature. 250 μL of an F-actin sample mix were prepared by incubating F-actin with MiuA for 30 min in PolyMix (100 mM KCl, mM MgCl_2_, 1 mM ATP, 10 mM imidazole pH 7.4) at room temperature. The respective F-actin binding protein was then added and incubated for 1 h at room temperature (the molar ratio of F-actin, MiuA (or jasplakinolide) and actin binding protein was 1:10:1). After incubation, 40 μL of the sample mix were prepared for a total SDS-PAGE sample. Pelleting of F-actin binding proteins was achieved by spinning the rest of the sample at 100,000 × g, at 4 °C for 1 h. 40 μL of the supernatant were prepared as a sample for SDS-PAGE. The pellets were dissolved in 200 μL of 1 × SDS-sample buffer, boiled and then each SDS-sample (total, supernatant and pellet) was loaded onto a SDS-PAGE gel separately to observe F-actin binding after electrophoresis. The gels were captured by using a ChemiDoc Imaging System (Bio-Rad Laboratories GmbH). The amounts of each protein were quantified using the Image Lab 6.0 Software.

### Assessment of the transcriptome

HUVECS at 80% confluency were treated with equipotent concentrations of MiuA or jasplakinolide (60 nM and 120 nM respectively) for 4 h. The concentrations were chosen in order to stay below levels causing visible alterations of cell morphology and overall actin structure. mRNA was cleaned up from cell lysates with Sera-Mag carboxylated magnetic beads (Thermo Fisher, Waltham, MA, USA) and reversely transcribed using a slightly modified SCRB-seq protocol^41^. During reverse transcription, sample-specific barcodes and unique molecular identifiers were incorporated into first strand cDNA. Next, samples were pooled and excess primers digested by Exonuclease I (Thermo Fisher, Waltham, MA, USA). cDNA was preamplified using KAPA HiFi HotStart polymerase (KAPA Biosystems). Sequencing libraries were constructed from cDNA using the Nextera XT Kit (Illumina, San Diego, CA, USA). Resulting libraries were quantified and sequenced at 10 nM on a HiSeq1500 (Illumina, San Diego, CA, USA). To obtain gene-wise expression values, raw sequencing data was processed using the zUMIs pipeline [34] using the Human genome build hg19 and Ensembl gene models (GRCh37.75). Transcriptome analysis was performed using the free statistical software R (v. 3.4.2). DESeq2 package (v.1.16.1) was used for normalization and differential expression (DE) analysis. DESeq2 models transcriptional count data using negative binomial distribution. Additional filtering was done using HTSFilter (v.1.16.0) to remove constant, lowly expressed genes. The final gene set consisted of 15 232 genes.

DE testing was based on Wald test. Multiple testing was accounted for by applying a global false discovery rate (FDR) correction to all comparisons. All genes with FDR < 0.1 were considered significant.

Enrichment analysis for Gene Ontology terms was performed using topGO package (v.2.28.0), specifying the ontology of Biological Processes (BP). Fisher’s exact test was applied to measure the significance of enrichment.

### Pyrene assay

Pyrene actin was purchased from Hypermol (Bielefeld, Germany) and diluted to a 1 mg/mL (24 μM) stock solution. Before use, spontaneously formed actin aggregates were removed by ultracentrifugation for 1 h at 40,000 rpm and 4 °C. 50 μL samples for the pyrene assay consisted of: 30 μL H_2_O, 10 μL 10 mM MgCl_2_ or 250 mM KCl, 5 μL F-actin Buffer (100 mM Imidazole-Cl pH 7.4, 10 mM ATP, Hypermol, Germany) as well as 5 μL DMSO (containing the indicated concentrations of MiuA). 10 μL pyrene actin (end concentration 1 µM) were rapidly added to start polymerization. Pyrene fluorescence was monitored at 400 nm every 20 s over 1 h in a 96-well fluorescence plate reader (Tecan) with 360 nm excitation.

### TIRF assays

Flow cells containing 15–20 μL of fluid were prepared as a sandwich of a cover slip (22 × 22 mm), two parafilm strips forming an approximately 5 mm wide channel, and a glass microscope slide (76 × 26 mm).

Atto488-actin and actin (unlabeled) from rabbit skeletal muscle were purchased from Hypermol (Bielefeld, Germany). Labeled actin was prepared by mixing Atto488-actin and actin 1:1 v/v. A stock solution of α-actinin from turkey gizzard smooth muscle (Hypermol, Bielefeld, Germany) was prepared by adding 1 mL H_2_O to the tube with α-actinin to obtain a working stock of 1 mg/mL. α-actinin was used as tethering protein for actin filaments, since it has turned out to alter filament structure and dynamics least^42^.

#### Nucleation and polymerization assay

Freshly prepared flow cells were first incubated with 25 μL 1% (w/v) bovine serum albumin (BSA). After 10 min, 25 μL α-actinin (1 mg/mL) was applied into the flow cell and incubated for 5 min. In the meantime, labeled actin (10 μM) was incubated 1:1 with 1/10 volume of 10 × Mg exchange buffer (400 μM MgCl_2_, 2 mM EGTA) and 1:8 with G-buffer (2 mM Tris-HCl, pH 8.0, 0.2 mM CaCl_2_, 0.5 mM DTT, 0.2 mM ATP, final pH 7.8) for 5 min on ice to convert Ca-ATP-actin to Mg-ATP-actin. Flow cells were then washed with 30 μL of G-buffer. 2 × Mg-ATP-actin (1 μM) was mixed 1:1 with 2 × TIRF buffer (100 mM KCl, 2 mM MgCl_2_, 2 mM EGTA, 30 mM imidazole, 30 mM D-glucose, 40 μg/mL catalase, 400 μg/mL glucose oxidase, 1% methylcellulose, 2% β-mercaptoethanol, final pH 7.4) containing MiuA as indicated, and the polymerization started. 30 μL of polymerizing actin were immediately loaded into the flow cell chamber and placed on the TIRF microscope (Leica Microsystems, Wetzlar, Germany) to start image acquisition. The amount of actin nuclei present in each frame was analyzed using programs custom-written in MATLAB (The MathWorks, Natick, MA) R2017a. For polymerization assays, fluorescence image sequences of actin polymerization were taken every 1 s for 5 min. Elongation rates were calculated by Image J software (version 1.49).

#### Depolymerization assay

Labeled F-actin was obtained by incubating labeled actin (10 μM) 1:1 with 1/10 volume of 10 × polymerization buffer and 1:8 with G-buffer for 1 h at room temperature. 20 μL 1:4 diluted F-actin (with F-buffer: 10 × polymerization buffer diluted 1:9 (v/v) with G-buffer) were loaded into a flow cell previously blocked with 1% BSA and coated with 1 mg/mL α-actinin. Then, 50 μL of 1 × TIRF buffer with 5 µΜ MiuA were gently applied into the flow chamber. The sample was immediately placed on the TIRF microscope and data acquisition was started. Actin depolymerization was followed in time by collecting individual frames every 15 s over 90 min.

#### Phalloidin competition assay

Rhodamine-phalloidin was purchased from Invitrogen. For the phalloidin competition assay, labeled F-actin (prepared as described above) was loaded into a freshly coated flow cell. TIRF buffer with 16.5 nM rhodamine-phalloidin and different concentrations of MiuA were applied as indicated into the flow chamber and immediately placed on the TIRF microscope to start image acquisition. Different regions of interest were measured. Fluorescence from the actin filaments were recorded in the green channel while the signal from rhodamine-labeled phalloidin was monitored in the red channel. The change in intensity of the rhodamine-labeled phalloidin (Δintensity) and the IC 50 of the displacement reaction were calculated using ImageJ software (version 1.49) and Prism software, respectively.

### Fluorescence correlation spectroscopy

FCS experiments were performed on a home-built confocal fluorescence set-up. LabTek slides were incubated with 10% BSA for 10 min. Atto488 labeled actin (99% labeled, Hypermol, Bielefeld, Germany) was incubated in Mg exchange buffer for 5 min on ice, then polymerization was started by adding KMEI buffer (100 mM KCl, 2 mM MgCl_2_, 2 mM EGTA, 30 mM imidazole) to a final actin concentration of 50 or 100 nM. FCS measurements were taken for 1 h in 10 min time intervals. FCS curves were plotted using PAM (PIE analysis with MATLAB, The MathWorks, MA, USA). The spike occurrence and intensity were analyzed using self-written code in MATLAB. A peak was considered a spike when the intensity was higher than the mean count rate of the measurement plus 4 times the standard deviation^13^.

### Docking studies

#### Structure Preparation

The MiuA structure was built using the Avogadro program^43^. The actin structure was taken from PDB-ID 1NWK^44^, where actin was crystallized in the presence of an ATP molecule and calcium ions. ATP was converted into ADP and the calcium ion was kept within the model system. The actin trimer was built using the structure from the PDB code 3J8A^45^ as a scaffold. Three replicas of the miuraenamide A bound monomer complexes were placed into this scaffold (see below for the complex formation). Missing hydrogen atoms of the actin proteins and ADP were added using the tleap module of the Amber14/AmberTools15^46^ program package. Each molecular system prepared in this work was solvated within a box of TIP3P^47^ water molecules by applying a 12 Å buffer region.

#### Parameters

The ff03^48^ force field parameters were chosen for the protein atoms. The ADP parameters by Carlson and coworkers^49^ were used. For the ligands, the General Amber Force Field (GAFF) parameters^50^ were chosen and the charges were calculated using the following stepwise strategy to obtain a precise set of atomic point charges: (i) Initially, atomic charges were calculated using the AM1 level and the bcc model as implemented in the antechamber module of AmberTools15, (ii) Molecular dynamics simulations were performed for 500 ns at 300 K in gas phase using these initial charges, (iii) the structures along the trajectory were clustered and the representative conformations of each cluster were optimized using the Gaussian09 program package (Gaussian Inc., Wallingford, CT, USA) (HF/6–31 G(d,p) level of theory) (iv) the electrostatic potential of the energetically most favorable conformation was calculated at the B3LYP/cc-pVTZ level of theory and the point charges were derived using the RESP procedure.

#### Molecular dynamics simulations

The same minimization and heat up procedure as described in our previous publications^51,52^ was applied to all of the modeled systems. The box size was gradually adjusted trough a sequential series of energy minimizations performed with the sander module of Amber to reach a target density of 1.0 g/cm^3^. The systems were first heated up from 0 to 50 K by applying a 3.0 kcal.mol^−1^.Å^−2^ restraint to the Cartesian position of all protein non-hydrogen atoms. The constraint was then reduced to affect only the backbone atoms while heating the system from 50 to 200 K. All restraints were then released while the temperature was increased to 300 K. The SHAKE algorithm^53^ was used to constraint all bonds involving hydrogen atoms. Long-range electrostatic interactions were calculated using the standard settings of the particle mesh Ewald method^54^. Periodic boundary conditions were applied to each system. Temperature and pressure were controlled by the Langevin thermostat (collision frequency of 4.0 ps^−1^) and Berendsen barostat (default Amber settings), respectively. The integration time step was set to 1 fs and a non-bonded cutoff of 12 Å was applied for non-bonded interactions. The simulations were performed using the cuda-enabled graphics processing units (GPUs) version of the pmemd module of Amber14^55^. After equilibration (see above) simulations were performed for 25 ns and 150 ns, for the MiuA bound actin monomer and for the MiuA bound trimer (holo), respectively. After building the MiuA bound structure on the F-actin scaffold (see Structure Preparation), the three ligands were removed from the complex (apo) and a 150 ns long MD simulation was run for this system as a control.

#### Conformational sampling of miuraenamide

MiuA was solvated in a box of water using the same protocol as introduced above. A protocol similar to that described above was used for heating up the system, however slightly modified to reach a target temperature of 370 K (i.e., two steps were added to the temperature ramp: 340 K and 370 K). Three parallel molecular dynamics simulations of 250 ns each were performed for each ligand at 370 K, starting from a different set of random velocities, thus reaching 750 ns of sampling per ligand. The conformations of each ligand were clustered along their respective 750 ns sampling trajectory using a RMSD-based hierarchical clustering as implemented in cpptraj with a target number of five clusters.

#### Molecular docking calculations

Broad sampling and subsequent refinement of the binding positions were performed using the DynaDock procedure as implemented in our in-house modeling program (DynaCell)^56^. As the binding site of the ligand is unknown, five distinct binding sites were tested. These sites were chosen based on a combination of experimental information obtained in the current study, prior knowledge on binding site of other examples of the actin stabilizing macrocyles^57^ and surface based visual analysis done by pymol. Five representative conformations of the ligands were used as starting structures for docking at each of the five chosen binding sites. The internal structure of the ligands was kept fixed during the broad sampling while the translational and rotational degrees of freedom of the ligand within the binding pocket were flexible, allowing, a maximum translation step of 2 Å (in all three Cartesian directions) and a rotational angle step of 30°. The van der Waals radii of the receptor and ligand atoms were allowed 80% of overlap to implicitly treat potential induced fit-based adaptations within the binding site^56^. The sampling was continued until 2000 poses were collected. These 2000 poses were further analyzed based on the overall position of the ligand (within a 15 Å radius from the active site) and its relative position to the other poses (maximum distance of 4 Å). This analysis yielded approximately 250 poses, which were further refined using a softcore-based potential and specialized MD algorithm (OPMD), as implemented in DynaCell. OPMD simulations were performed for 20 ps using backbone restraints with a force constant of 100 kJ mol^−1^ nm^−2^ ^56^. The side chain atoms of the protein and all atoms of the ligands remained free for flexible conformational adaption of the binding site to the sampled ligand pose (induced-fit phenomenon). Structures without any remaining van der Waals overlap were characterized as successful poses and a total of approximately 400 refined poses were obtained for each of the five starting conformations of the ligand in each of the five explored binding sites. Afterwards, the resulting poses were clustered based on their RMSD (only non-hydrogen atoms) with a maximum distance of 2 Å between the clusters. Following the clustering analysis, the two best-ranked poses from the first three clusters were selected, if three or more different cluster were observed. This detailed protocol (including both broad sampling and the refinement) was applied to model each of the five possible binding sites (M1-M5). Combining the chemical interaction analysis (as detailed in the Results section) with the short stability analysis (results not shown), the binding site M1 was chosen as the most probable binding site and further calculations were performed on the complex with MiuA bound in this region.

#### Binding energy calculations

Each binding mode was equilibrated for 5 ns using the same MD scheme as above. Three distinct simulations (each starting with different velocities) were performed on equilibrated structures for further 15 ns. The time step was 1 fs. A total of 225,000 complex frames (3 × 75,000) were saved during 15 ns simulation. The binding free energies were estimated using the Molecular Mechanics-Generalized Born Surface Area (MMGBSA) approach^58^ by means of the MMGBSA.py module^59^ of Amber16/AmberTools17^60^. The contribution of the solvent was computed using the default Generalized Born Surface Area (GBSA) options of the script with a probe radius of 1.4 Å and the ‘mbondi2’ radii set^61^.

### Statistical analysis

Quantitative data are expressed as mean ± SEM. Statistical analysis was performed using the software GraphPad Prism Version 7.02 (GraphPad Software, Inc., La Jolla, CA, USA). Statistical differences were evaluated by using Kruskal–Wallis test or one-way analysis of variance (ANOVA). P-values less than 0.05 were considered to be significant. For all tests, three replicates from three independent experiments (n = 3) were used. Specific information on the statistical procedures used can be found in the respective figure legends.

## Supplementary information

Supplementary Information

## Data Availability

The datasets generated during and/or analysed during the current study are available from the corresponding author on reasonable request.

## References

[1] Katagiri K, Matsuura S (1971). Antitumor activity of cytochalasin D. J Antibiot (Tokyo).

[2] Low I, Dancker P, Wieland T (1975). Stabilization of F-actin by phalloidin. Reversal of the destabilizing effect of cytochalasin B. FEBS Lett.

[3] Spector I, Shochet NR, Kashman Y, Groweiss A (1983). Latrunculins: novel marine toxins that disrupt microfilament organization in cultured cells. Science.

[4] Bubb MR, Senderowicz AM, Sausville EA, Duncan KL, Korn ED (1994). Jasplakinolide, a cytotoxic natural product, induces actin polymerization and competitively inhibits the binding of phalloidin to F-actin. J Biol Chem.

[5] Davidson AJ, Wood W (2016). Unravelling the Actin Cytoskeleton: A New Competitive Edge?. Trends Cell Biol.

[6] Goldschmidt-Clermont PJ (1992). The control of actin nucleotide exchange by thymosin beta 4 and profilin. A potential regulatory mechanism for actin polymerization in cells. Mol Biol Cell.

[7] Miralles F, Posern G, Zaromytidou AI, Treisman R (2003). Actin dynamics control SRF activity by regulation of its coactivator MAL. Cell.

[8] Klenchin VA (2003). Trisoxazole macrolide toxins mimic the binding of actin-capping proteins to actin. Nat Struct Biol.

[9] Karmann L, Schultz K, Herrmann J, Müller R, Kazmaier U (2015). Total syntheses and biological evaluation of miuraenamides. Angewandte Chemie International Edition.

[10] Sumiya E (2011). Cell-morphology profiling of a natural product library identifies bisebromoamide and miuraenamide A as actin filament stabilizers. ACS chemical biology.

[11] Moser C (2017). Persistent inhibition of pore-based cell migration by sub-toxic doses of miuraenamide, an actin filament stabilizer. Sci Rep.

[12] Lee E, Shelden EA, Knecht DA (1998). Formation of F-actin aggregates in cells treated with actin stabilizing drugs. Cell Motil Cytoskeleton.

[13] Crevenna AH (2013). Electrostatics control actin filament nucleation and elongation kinetics. J Biol Chem.

[14] Mentes A (2018). High-resolution cryo-EM structures of actin-bound myosin states reveal the mechanism of myosin force sensing. Proc Natl Acad Sci USA.

[15] Merino F (2018). Structural transitions of F-actin upon ATP hydrolysis at near-atomic resolution revealed by cryo-EM. Nat Struct Mol Biol.

[16] Galkin VE (2011). Remodeling of actin filaments by ADF/cofilin proteins. Proc Natl Acad Sci USA.

[17] Dominguez R (2004). Actin-binding proteins–a unifying hypothesis. Trends Biochem Sci.

[18] Povarova OI, Uversky VN, Kuznetsova IM, Turoverov KK (2014). Actinous enigma or enigmatic actin: Folding, structure, and functions of the most abundant eukaryotic protein. Intrinsically Disord Proteins.

[19] Xue B, Robinson RC (2013). Guardians of the actin monomer. Eur J Cell Biol.

[20] Coutts AS, La Thangue NB (2015). Actin nucleation by WH2 domains at the autophagosome. Nat Commun.

[21] Kast DJ, Zajac AL, Holzbaur EL, Ostap EM, Dominguez R (2015). WHAMM Directs the Arp2/3 Complex to the ER for Autophagosome Biogenesis through an Actin Comet Tail Mechanism. Curr Biol.

[22] Suarez C, Kovar DR (2016). Internetwork competition for monomers governs actin cytoskeleton organization. Nat Rev Mol Cell Biol.

[23] Tanaka J (2003). Biomolecular mimicry in the actin cytoskeleton: mechanisms underlying the cytotoxicity of kabiramide C and related macrolides. Proc Natl Acad Sci USA.

[24] Waldmann H (2008). Total synthesis of chondramide C and its binding mode to F-actin. Angew Chem Int Ed Engl.

[25] Iizuka T (2006). Miuraenamides A and B, novel antimicrobial cyclic depsipeptides from a new slightly halophilic myxobacterium: taxonomy, production, and biological properties. The Journal of antibiotics.

[26] Gegenfurtner Florian A., Zisis Themistoklis, Al Danaf Nader, Schrimpf Waldemar, Kliesmete Zane, Ziegenhain Christoph, Enard Wolfgang, Kazmaier Uli, Lamb Don C., Vollmar Angelika M., Zahler Stefan (2018). Transcriptional effects of actin-binding compounds: the cytoplasm sets the tone. Cellular and Molecular Life Sciences.

[27] Senderowicz AM (1995). Jasplakinolide’s inhibition of the growth of prostate carcinoma cells *in vitro* with disruption of the actin cytoskeleton. J Natl Cancer Inst.

[28] Hayot C (2006). Characterization of the activities of actin-affecting drugs on tumor cell migration. Toxicol Appl Pharmacol.

[29] Wu L (2016). Sinoporphyrin sodium mediated photodynamic therapy inhibits the migration associated with collapse of F-actin filaments cytoskeleton in MDA-MB-231 cells. Photodiagnosis Photodyn Ther.

[30] Virtanen JA, Vartiainen MK (2017). Diverse functions for different forms of nuclear actin. Curr Opin Cell Biol.

[31] Pospich S (2017). Near-atomic structure of jasplakinolide-stabilized malaria parasite F-actin reveals the structural basis of filament instability. Proc Natl Acad Sci USA.

[32] Tannert R (2010). Synthesis and structure-activity correlation of natural-product inspired cyclodepsipeptides stabilizing F-actin. J Am Chem Soc.

[33] Easter DC, Terrell DA, Roof JA (2005). Monte Carlo studies of isomers, structures, and properties in benzene-cyclohexane clusters: computation strategy and application to the dimer and trimer, (C6H6)(C6H12)n, n = 1-2. J Phys Chem A.

[34] Lanzarotti E, Biekofsky RR, Estrin DA, Marti MA, Turjanski AG (2011). Aromatic-aromatic interactions in proteins: beyond the dimer. J Chem Inf Model.

[35] Tauer TP, Sherrill CD (2005). Beyond the benzene dimer: an investigation of the additivity of pi-pi interactions. J Phys Chem A.

[36] Engkvist O, Hobza P, Selzle HL, Schlag EW (1999). Benzene trimer and benzene tetramer: Structures and properties determined by the nonempirical model (NEMO) potential calibrated from the CCSD(T) benzene dimer energies. The Journal of Chemical Physics.

[37] Krause H, Ernstberger B, Neusser HJ (1991). Binding energies of small benzene clusters. Chemical Physics Letters.

[38] Wiggan O, Schroder B, Krapf D, Bamburg JR, DeLuca JG (2017). Cofilin Regulates Nuclear Architecture through a Myosin-II Dependent Mechanotransduction Module. Sci Rep.

[39] Munsie LN, Desmond CR, Truant R (2012). Cofilin nuclear-cytoplasmic shuttling affects cofilin-actin rod formation during stress. J Cell Sci.

[40] Fiore A (2017). Laminin-111 and the Level of Nuclear Actin Regulate Epithelial Quiescence via Exportin-6. Cell Rep.

[41] Parekh, S., Ziegenhain, C., Vieth, B., Enard, W. & Hellmann, I. zUMIs - A fast and flexible pipeline to process RNA sequencing data with UMIs. *Gigascience* **7**, 10.1093/gigascience/giy059 (2018). 10.1093/gigascience/giy059 PMC6007394 29846586

[42] Crevenna, A. H. *et al*. Side-binding proteins modulate actin filament dynamics. *Elife* **4**, 10.7554/eLife.04599 (2015). 10.7554/eLife.04599 PMC4375888 25706231

[43] Hanwell MD (2012). Avogadro: an advanced semantic chemical editor, visualization, and analysis platform. J Cheminform.

[44] Graceffa P, Dominguez R (2003). Crystal structure of monomeric actin in the ATP state. Structural basis of nucleotide-dependent actin dynamics. J Biol Chem.

[45] von der Ecken J (2015). Structure of the F-actin-tropomyosin complex. Nature.

[46] Case, D. A. *et al*. AMBER 2015. *University of California*, *San Francisco* (2015).

[47] Jorgensen WL, Chandrasekhar J, Madura JD, Impey RW, Klein ML (1983). Comparison of simple potential functions for simulating liquid water. The Journal of Chemical Physics.

[48] Duan Y (2003). A point-charge force field for molecular mechanics simulations of proteins based on condensed-phase quantum mechanical calculations. J Comput Chem.

[49] Meagher KL, Redman LT, Carlson HA (2003). Development of polyphosphate parameters for use with the AMBER force field. J Comput Chem.

[50] Wang J, Wolf RM, Caldwell JW, Kollman PA, Case DA (2004). Development and testing of a general amber force field. J Comput Chem.

[51] Marcinowski M (2013). Conformational selection in substrate recognition by Hsp70 chaperones. J Mol Biol.

[52] Schneider M (2016). BiPPred: Combined sequence- and structure-based prediction of peptide binding to the Hsp70 chaperone BiP. Proteins.

[53] Ryckaert J-P, Ciccotti G, Berendsen HJC (1977). Numerical integration of the cartesian equations of motion of a system with constraints: molecular dynamics of n-alkanes. Journal of Computational Physics.

[54] Essmann U (1995). A smooth particle mesh Ewald method. The Journal of Chemical Physics.

[55] Götz AW (2012). Routine Microsecond Molecular Dynamics Simulations with AMBER on GPUs. 1. Generalized Born. Journal of Chemical Theory and Computation.

[56] Antes I (2010). DynaDock: A new molecular dynamics-based algorithm for protein-peptide docking including receptor flexibility. Proteins.

[57] Allingham JS, Klenchin VA, Rayment I (2006). Actin-targeting natural products: structures, properties and mechanisms of action. Cell Mol Life Sci.

[58] Srinivasan J, Cheatham TE, Cieplak P, Kollman PA, Case DA (1998). Continuum solvent studies of the stability of DNA, RNA, and phosphoramidate-DNA helices. J. Am. Chem. Soc..

[59] Miller BR (2012). MMPBSA. py: an efficient program for end-state free energy calculations. J. Chem. Theory Comput..

[60] Case, D. *et al*. AMBER 17. *University of California: San Francisco*, *CA*, *USA* (2017).

[61] Srinivasan J, Trevathan MW, Beroza P, Case DA (1999). Application of a pairwise generalized Born model to proteins and nucleic acids: inclusion of salt effects. Theoretical Chemistry Accounts: Theory, Computation, and Modeling (Theoretica Chimica Acta).

