# Supplementary material for: Actin stabilizing compounds show specific biological effects due to their binding mode
Source: Sci Rep. 2019 Jul 5;9:9731. doi: 10.1038/s41598-019-46282-w (PMC6611809; doi:10.1038/s41598-019-46282-w)
Supplement: Supplementary file 1 — Supplementary Information [file 41598_2019_46282_MOESM1_ESM.pdf]

Supplementary Information

Actin stabilizing compounds show specific biological effects due to their binding mode

Shuaijun Wang, Alvaro H. Crevenna, Ilke Ugur, Antoine Marion, Iris Antes, Uli Kazmaier, Maria Hoyer, Don C. Lamb, Florian Gegenfurtner, Zane Kliesmete, Christoph Ziegenhain, Wolfgang Enard, Angelika Vollmar and Stefan Zahler

Supplementary Table 1. *List of the genes that are differently regulated in cells treated with miuraenamide A vs. jasplakinolide*

Supplementary Table 2. *List of the gene ontologies (GO) that are significantly regulated by MiuA treatment*

Supplementary Table 3. *List of significant gene set enrichment (category "biological process", Fisher's exact test: <0.01) based on differentially regulated genes by jasp treatment compared to control*

Supplementary Figure 1. *Control experiments for the G-actin binding assay. (a) Gelsolin does not bind to beads that are not coupled to actin, and binding of gelsolin depends on the presence of* Ca^{2+}*. (b) Gelsolin competes with cofilin for binding to G-actin. (c) Miu inhibits binding of cofilin to G-actin also at a molar ratio of 1:1.*

Supplementary Figure 2. *Confocal images of F-actin (labelled with rhodamine-phalloidin, red) and cofilin (Alexa488, green) of untreated control cells (a) and cells treated with 50 nM MiuA for 30 min (b). After treatment with MiuA cofilin co-localizes less with F-actin as also seen in the fluorescence intensity profiles along the indicated lines of the two channels, respectively.*

Supplementary Table 1

ENSEMBL	SYMBOL	baseMean	log2FoldChange	pvalue	padj
ENSG00000143869	GDF7	12,1612956	3,211230417	0,00066079	0,04390488
ENSG00000117707	PROX1	6,9950098	1,200773712	0,00038769	0,02984357
ENSG00000254409	NA	6,75172368	1,031068621	0,00075856	0,04826523
ENSG00000146592	CREB5	4,81387076	0,989692802	0,00214647	0,09695071
ENSG00000134070	IRAK2	13,4395225	0,85821141	0,00065576	0,04361793
ENSG00000138764	CCNG2	13,0686991	0,847376617	0,00013811	0,01398979
ENSG00000159167	STC1	22,7951994	0,837013708	1,71E-05	0,00295321
ENSG00000130522	JUND	41,7888712	0,767387825	3,31E-08	1,97E-05
ENSG00000196843	ARID5A	23,5057975	0,762749431	6,30E-06	0,00137834
ENSG00000147324	MFHAS1	15,1412852	0,732925029	0,00140356	0,0727488
ENSG00000185262	UBALD2	42,8586666	0,64487482	3,38E-07	0,00013123
ENSG00000131773	KHDRBS3	23,5340208	0,627094638	0,00023705	0,02070649
ENSG00000176907	C8orf4	26,9181463	0,616227006	6,12E-05	0,00760694
ENSG00000122861	PLAU	58,8854195	0,594281362	5,34E-07	0,0001904
ENSG00000102802	MEDAG	20,0934396	0,590968616	0,00142928	0,07353533
ENSG00000156030	ELMSAN1	64,7755138	0,572897126	8,00E-06	0,00166579
ENSG00000073756	PTGS2	36,0905195	0,570224831	7,02E-05	0,00836912
ENSG00000267519	LOC284454	41,4665223	0,560945617	6,59E-05	0,00792582
ENSG00000130513	GDF15	708,008436	0,517880532	1,74E-13	3,36E-10
ENSG00000114796	KLHL24	27,0924735	0,509324298	0,00116119	0,06431738
ENSG00000161940	BCL6B	43,7222369	0,50318591	6,45E-05	0,00786805
ENSG00000215788	TNFRSF25	32,6352593	0,496333564	0,00065528	0,04360987
ENSG00000102858	MGRN1	29,1883624	0,471525312	0,0018171	0,08659549
ENSG00000165434	PGM2L1	36,2370469	0,451787136	0,00092966	0,05523594
ENSG00000104419	NDRG1	65,4743738	0,433732745	5,07E-05	0,00656209
ENSG00000008513	ST3GAL1	52,2455518	0,40094068	0,00046526	0,03407745
ENSG00000173575	CHD2	47,917017	0,399692546	0,00113307	0,06351039
ENSG00000106144	CASP2	37,0511503	0,383660036	0,00149717	0,07580822
ENSG00000144580	CNOT9	90,3178913	0,37166965	3,39E-05	0,00489103
ENSG00000164938	TP53INP1	63,920527	0,370973762	0,00037737	0,0291967
ENSG00000171056	SOX7	54,8476519	0,360656701	0,00072401	0,04700333
ENSG00000137834	SMAD6	121,948355	0,3585581	0,0002086	0,01893161
ENSG00000130340	SNX9	66,2626525	0,355227145	0,00050724	0,03650922
ENSG00000138434	SSFA2	54,9343108	0,344254459	0,00101624	0,05874504
ENSG00000107438	PDLIM1	107,23865	0,332260672	0,00053902	0,03816533
ENSG00000197632	SERPINB2	78,299862	0,326985741	0,00160777	0,07936332
ENSG00000136802	LRRC8A	235,319363	0,325326803	1,92E-08	1,23E-05
ENSG00000183578	TNFAIP8L3	90,7877088	0,315874037	0,00036511	0,02855605
ENSG00000228470	NA	89,6018438	0,312031431	0,00029326	0,02442774
ENSG00000158769	F11R	82,0601165	0,302235223	0,00139272	0,07246435
ENSG00000151012	SLC7A11	82,7056378	0,301238012	0,00127783	0,06819069
ENSG00000087074	PPP1R15A	106,256495	0,27161909	0,00072546	0,04702655
ENSG00000213707	NA	140,893306	0,268136758	0,00222089	0,0992403
ENSG00000011422	PLAUR	148,981947	0,25625363	0,0005689	0,03952355
ENSG00000157191	NECAP2	119,240373	0,237760585	0,00166523	0,08146062
ENSG00000162407	PLPP3	172,539066	0,233707345	0,00183159	0,08711557
ENSG00000134294	SLC38A2	357,910122	0,232223562	0,00010455	0,01136282
ENSG00000124762	CDKN1A	319,443467	0,228764937	2,38E-05	0,00375222
ENSG00000229344	NA	5665,93474	0,211642945	9,01E-05	0,0100968

ENSG00000067082	KLF6	237,579626	0,193719487	0,00115517	0,06422145
ENSG00000124766	SOX4	1254,7448	0,18198231	0,00222527	0,09932691
ENSG00000131711	MAP1B	576,803941	0,176298073	3,93E-05	0,00547202
ENSG00000142192	APP	351,095286	0,162731651	0,00198436	0,0917555
ENSG00000198786	ND5	838,781205	0,150652582	0,00074444	0,04771955
ENSG00000125810	CD93	386,776359	0,146201722	0,00204198	0,0933214
ENSG00000151131	C12orf45	268,382041	-0,21024775	0,00214708	0,09695071
ENSG00000138385	SSB	185,094093	-0,214006338	0,00142173	0,07334747
ENSG00000149357	LAMTOR1	139,431261	-0,239179547	0,00079173	0,04957737
ENSG00000133226	SRRM1	375,102906	-0,272147429	0,00051291	0,0367654
ENSG00000115875	SRSF7	119,235007	-0,297525344	0,00020567	0,01881552
ENSG00000139168	ZCRB1	131,131784	-0,304510218	0,0014063	0,07282885
ENSG00000133773	CCDC59	85,2291742	-0,307601768	0,00201229	0,09246211
ENSG00000238072	NA	69,5976404	-0,307819146	0,00215496	0,09707743
ENSG00000048162	NOP16	134,341148	-0,313659024	2,15E-05	0,00348392
ENSG00000240298	NA	62,3763571	-0,319876639	0,00191628	0,08974243
ENSG00000179131	NA	77,2328996	-0,331069114	0,00216514	0,09750007
ENSG00000164741	DLC1	51,9483834	-0,345891822	0,00185087	0,08762236
ENSG00000142871	CYR61	235,361041	-0,370878102	0,00016354	0,01591759
ENSG00000266086	NA	71,1093436	-0,392386662	5,06E-05	0,00655592
ENSG00000155850	SLC26A2	45,6745027	-0,444084894	0,000238	0,02074545
ENSG00000179144	GIMAP7	73,1894221	-0,469074649	4,46E-05	0,00597939
ENSG00000080608	PUM3	104,021307	-0,474190625	1,08E-05	0,0020823
ENSG00000099260	PALMD	77,5489432	-0,483968208	9,46E-06	0,00187135
ENSG00000237049	NA	30,5010276	-0,493060911	0,00108841	0,06171657
ENSG00000137463	MGARP	24,9273743	-0,521143856	0,00154892	0,077368
ENSG00000196873	CBWD3	26,5944043	-0,552697818	0,00045917	0,03376768
ENSG00000117152	RGS4	33,6028624	-0,589116209	0,00013311	0,01361863
ENSG00000120738	EGR1	15,7402692	-0,629288447	0,0019559	0,09096887
ENSG00000099860	GADD45B	22,2964557	-0,635534712	0,00030033	0,02486209
ENSG00000107984	DKK1	99,9401769	-0,650902736	6,50E-05	0,0078897
ENSG00000078401	EDN1	260,830597	-0,680275946	7,62E-09	5,73E-06
ENSG00000244756	NA	23,7608697	-0,767663412	1,30E-05	0,00241149
ENSG00000157168	NRG1	20,6776412	-0,778532334	2,79E-05	0,00420651
ENSG00000180104	EXOC3	15,8029714	-0,797772381	0,00011715	0,01231661
ENSG00000125378	BMP4	24,0942604	-0,826740033	6,51E-05	0,0078897
ENSG00000124523	SIRT5	8,94292235	-0,852466902	0,0010919	0,06182819
ENSG00000118523	CTGF	679,769853	-0,85885136	1,07E-14	2,38E-11
ENSG00000148677	ANKRD1	3885,6597	-0,889321614	9,17E-13	1,55E-09
ENSG00000178882	RFLNA	23,2346925	-1,022347951	3,40E-07	0,00013141
ENSG00000243537	NA	4,24821161	-1,286558947	0,00148006	0,07533573
ENSG00000128383	APOBEC3A	3,57717737	-1,405510384	0,00144089	0,07392863
ENSG00000171016	PYGO1	3,26208156	-1,439309644	0,00132005	0,06963448
ENSG00000218472	NA	5,56373608	-1,588729529	0,00032532	0,02634036
ENSG00000275757	LOC10000858	10,1139674	-1,765498882	2,05E-05	0,00338016
ENSG00000235332	NA	2,51970313	-1,860462883	0,00089551	0,05386374
ENSG00000109846	CRYAB	5,56763057	-2,016951635	5,58E-06	0,0012453
ENSG00000166165	CKB	9,31435082	-2,276836856	3,17E-05	0,00467047
ENSG00000250397	NA	1,89401328	-2,543100155	0,00187365	0,08825596
ENSG00000231034	NA	2,01281478	-2,603037911	0,00179789	0,08591501

ENSG00000234576	NA	7,74783861	-3,140189044	0,00080679	0,05013368
ENSG00000278806	NA	10,140733	-4,458140103	1,89E-05	0,00316203

Supplementary Table 2

GO,ID	Term	Annotated	Significant	Expected	Fisher
GO:0071260	cellular response to mechanical stimulus	56	11	2,87	0,00011
GO:0045987	positive regulation of smooth muscle contraction	11	5	0,56	0,00012
GO:0042273	ribosomal large subunit biogenesis	57	11	2,92	0,00012
GO:0045214	sarcomere organization	18	6	0,92	0,00019
GO:0030335	positive regulation of cell migration	265	31	13,59	0,0002
GO:0038096	Fc-gamma receptor signaling pathway involved in phagocytosis	62	11	3,18	0,00027
GO:0010592	positive regulation of lamellipodium assembly	13	5	0,67	0,00032
GO:0007015	actin filament organization	253	39	12,97	0,00036
GO:0045601	regulation of endothelial cell differentiation	20	6	1,03	0,00037
GO:0090151	establishment of protein localization to mitochondrial membrane	14	5	0,72	0,00047
GO:2001222	regulation of neuron migration	21	6	1,08	0,00049
GO:0030220	platelet formation	15	5	0,77	0,00068
GO:0030901	midbrain development	59	10	3,03	0,00075
GO:0002064	epithelial cell development	124	19	6,36	0,00081
GO:0032970	regulation of actin filament-based process	231	34	11,84	0,00092
GO:0007254	JNK cascade	130	16	6,67	0,00097
GO:0060996	dendritic spine development	61	10	3,13	0,00098
GO:0070423	nucleotide-binding oligomerization domain containing signaling pathw	32	7	1,64	0,00098
GO:0071347	cellular response to interleukin-1	51	9	2,61	0,00101
GO:0008361	regulation of cell size	107	14	5,49	0,00109
GO:0048146	positive regulation of fibroblast proliferation	33	7	1,69	0,00119
GO:0001503	ossification	240	24	12,31	0,00131
GO:0060333	interferon-gamma-mediated signaling pathway	53	9	2,72	0,00134
GO:0071417	cellular response to organonitrogen compound	341	31	17,48	0,00136
GO:0006954	inflammatory response	312	29	16	0,00137
GO:0034332	adherens junction organization	98	13	5,02	0,00143
GO:0007163	establishment or maintenance of cell polarity	123	15	6,31	0,00152
GO:0002181	cytoplasmic translation	44	8	2,26	0,00156
GO:0006937	regulation of muscle contraction	66	14	3,38	0,00166
GO:1900026	positive regulation of substrate adhesion-dependent cell spreading	26	6	1,33	0,00169
GO:0016601	Rac protein signal transduction	26	6	1,33	0,00169
GO:0007588	excretion	18	5	0,92	0,00171

GO:1902905	positive regulation of supramolecular fiber organization	120	18	6,15	0,00174
GO:0022618	ribonucleoprotein complex assembly	177	19	9,08	0,0018
GO:2000146	negative regulation of cell motility	154	18	7,9	0,00229
GO:0017148	negative regulation of translation	129	15	6,61	0,00245
GO:0002262	myeloid cell homeostasis	104	13	5,33	0,00246
GO:0051092	positive regulation of NF-kappaB transcription factor activity	93	12	4,77	0,00273
GO:0030260	entry into host cell	93	12	4,77	0,00273
GO:0035094	response to nicotine	20	5	1,03	0,00284
GO:0043123	positive regulation of I-kappaB kinase/NF-kappaB signaling	133	15	6,82	0,0033
GO:0071356	cellular response to tumor necrosis factor	187	19	9,59	0,00337
GO:0043542	endothelial cell migration	122	14	6,26	0,0038
GO:0051216	cartilage development	110	13	5,64	0,00404
GO:0030216	keratinocyte differentiation	62	9	3,18	0,00409
GO:0007179	transforming growth factor beta receptor signaling pathway	123	14	6,31	0,00409
GO:0000187	activation of MAPK activity	86	11	4,41	0,00432
GO:0034314	Arp2/3 complex-mediated actin nucleation	31	6	1,59	0,00434
GO:0061028	establishment of endothelial barrier	31	6	1,59	0,00434
GO:0048333	mesodermal cell differentiation	22	5	1,13	0,00442
GO:0010470	regulation of gastrulation	22	5	1,13	0,00442
GO:0003206	cardiac chamber morphogenesis	75	10	3,85	0,00474
GO:0045446	endothelial cell differentiation	74	15	3,79	0,00479
GO:0030048	actin filament-based movement	67	12	3,44	0,00503
GO:0002009	morphogenesis of an epithelium	341	29	17,48	0,00505
GO:1901185	negative regulation of ERBB signaling pathway	42	7	2,15	0,00505
GO:0008064	regulation of actin polymerization or depolymerization	123	20	6,31	0,00516
GO:0097421	liver regeneration	23	5	1,18	0,00542
GO:0030198	extracellular matrix organization	182	18	9,33	0,00565
GO:0001525	angiogenesis	269	28	13,79	0,00613
GO:0061842	microtubule organizing center localization	24	5	1,23	0,00656
GO:0030195	negative regulation of blood coagulation	24	5	1,23	0,00656

Supplementary Table 3

GO,ID	Term	Annotated	Significant	Expected	Fisher
GO:0030335	positive regulation of cell migration	265	14	4,39	0,00013
GO:0007015	actin filament organization	253	22	4,19	0,00014
GO:0034314	Arp2/3 complex-mediated actin nucleation	31	5	0,51	0,00014
GO:0007163	establishment or maintenance of cell polarity	123	9	2,04	0,0002
GO:0002576	platelet degranulation	80	7	1,33	0,00035
GO:0007229	integrin-mediated signaling pathway	61	6	1,01	0,00049
GO:0071346	cellular response to interferon-gamma	71	6	1,18	0,00111
GO:0003206	cardiac chamber morphogenesis	75	8	1,24	0,00116
GO:0006937	regulation of muscle contraction	66	8	1,09	0,00194
GO:0032956	regulation of actin cytoskeleton organization	210	17	3,48	0,00198
GO:0003014	renal system process	55	5	0,91	0,00209
GO:2000379	positive regulation of reactive oxygen species me	55	5	0,91	0,00209
GO:0045165	cell fate commitment	110	7	1,82	0,0023
GO:0048145	regulation of fibroblast proliferation	57	5	0,94	0,00246
GO:0019221	cytokine-mediated signaling pathway	320	13	5,3	0,00253
GO:0000910	cytokinesis	113	7	1,87	0,00268
GO:0030509	BMP signaling pathway	90	6	1,49	0,00374
GO:0032970	regulation of actin filament-based process	231	20	3,83	0,00375
GO:0051092	positive regulation of NF-kappaB transcription fac	93	6	1,54	0,00439
GO:0009612	response to mechanical stimulus	124	7	2,05	0,00449
GO:0002064	epithelial cell development	124	7	2,05	0,00449
GO:0048812	neuron projection morphogenesis	344	13	5,7	0,00468
GO:0032535	regulation of cellular component size	236	17	3,91	0,00547
GO:0051017	actin filament bundle assembly	98	6	1,62	0,00567
GO:0045860	positive regulation of protein kinase activity	312	12	5,17	0,00568
GO:0043588	skin development	130	7	2,15	0,00581
GO:0034333	adherens junction assembly	71	5	1,18	0,00634
GO:0002009	morphogenesis of an epithelium	341	17	5,65	0,00637
GO:0006936	muscle contraction	153	15	2,53	0,00639
GO:0030307	positive regulation of cell growth	103	6	1,71	0,0072
GO:0001525	angiogenesis	269	13	4,46	0,00737
GO:0071560	cellular response to transforming growth factor b	155	10	2,57	0,00753

GO:0045446	endothelial cell differentiation	74	5	1,23	0,00755
GO:0071158	positive regulation of cell cycle arrest	74	5	1,23	0,00755
GO:0007160	cell-matrix adhesion	139	7	2,3	0,00829
GO:0008360	regulation of cell shape	108	6	1,79	0,00901
GO:0001933	negative regulation of protein phosphorylation	292	11	4,84	0,00925
GO:0003231	cardiac ventricle development	78	7	1,29	0,00926
GO:0008217	regulation of blood pressure	78	5	1,29	0,00938

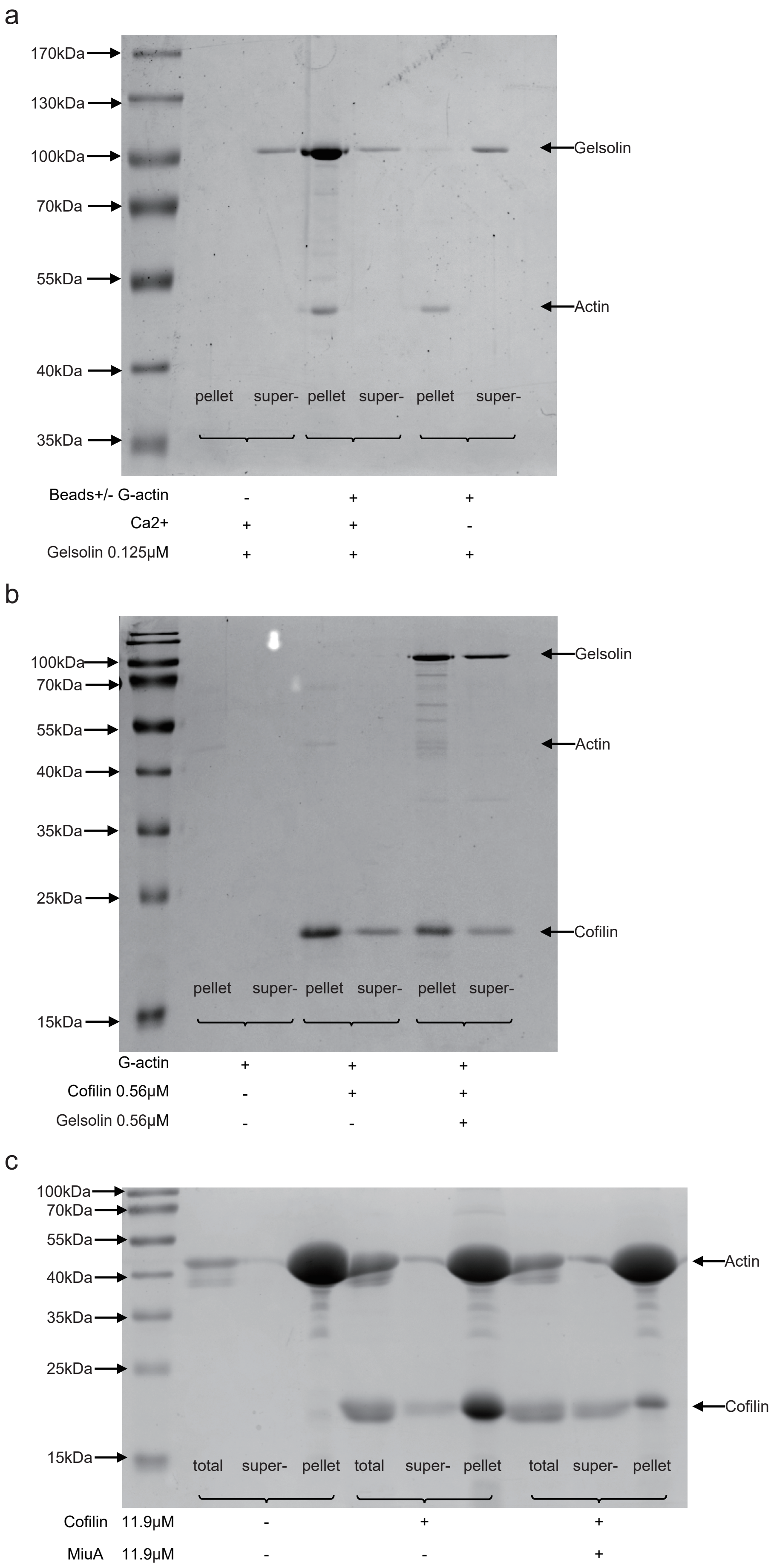

Supplementary Figure 1

a

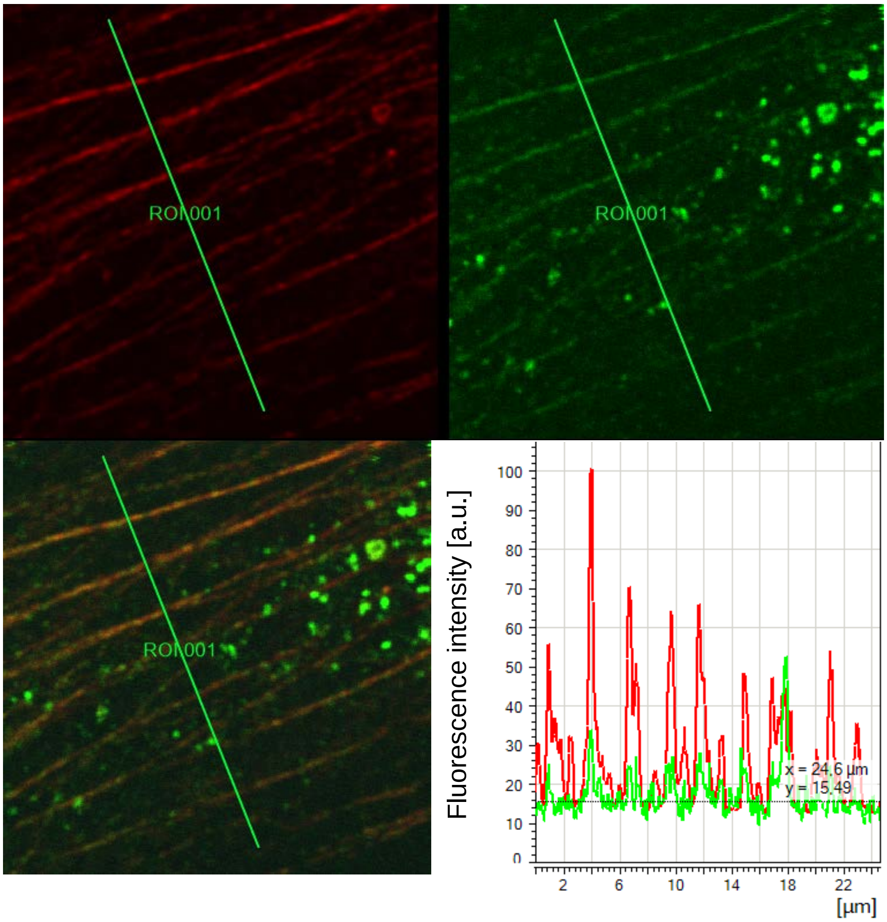

b

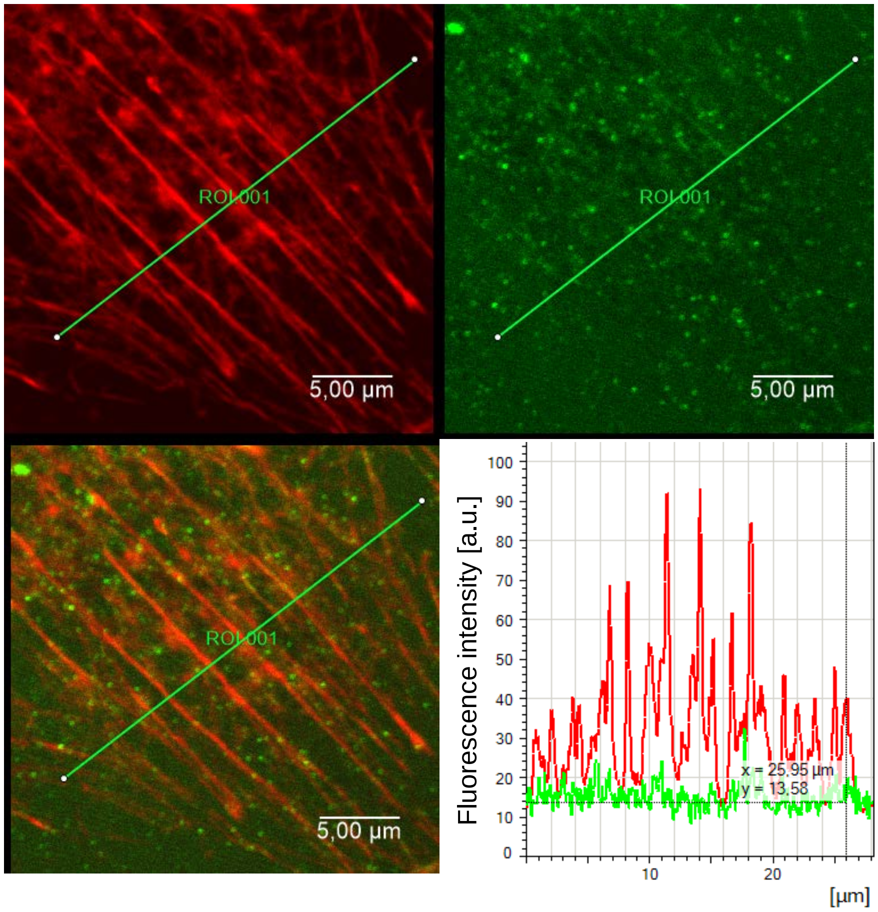

Supplementary Figure 2
